# Jackdaws form categorical prototypes based on experience with category exemplars

**DOI:** 10.1007/s00429-023-02651-w

**Published:** 2023-06-01

**Authors:** Aylin Apostel, Lukas Alexander Hahn, Jonas Rose

**Affiliations:** Neural Basis of Learning, Institute of Cognitive Neuroscience, Faculty of Psychology, Ruhr University Bochum, 44801 Bochum, Germany

**Keywords:** Categorization learning, RUBubbles, Avian cognition, Prototype- and exemplar-based, Variability

## Abstract

Categorization represents one cognitive ability fundamental to animal behavior. Grouping of elements based on perceptual or semantic features helps to reduce processing resources and facilitates appropriate behavior. Corvids master complex categorization, yet the detailed categorization learning strategies are less well understood. We trained two jackdaws on a delayed match to category paradigm using a novel, artificial stimulus type, RUBubbles. Both birds learned to differentiate between two session-unique categories following two distinct learning protocols. Categories were either introduced via central category prototypes (low variability approach) or using a subset of diverse category exemplars from which diagnostic features had to be identified (high variability approach). In both versions, the stimulus similarity relative to a central category prototype explained categorization performance best. Jackdaws consistently used a central prototype to judge category membership, regardless of whether this prototype was used to introduce distinct categories or had to be inferred from multiple exemplars. Reliance on a category prototype occurred already after experiencing only a few trials with different category exemplars. High stimulus set variability prolonged initial learning but showed no consistent beneficial effect on later generalization performance. High numbers of stimuli, their perceptual similarity, and coherent category structure resulted in a prototype-based strategy, reflecting the most adaptive, efficient, and parsimonious way to represent RUBubble categories. Thus, our birds represent a valuable comparative animal model that permits further study of category representations throughout learning in different regions of a brain producing highly cognitive behavior.

**Graphical abstract:**

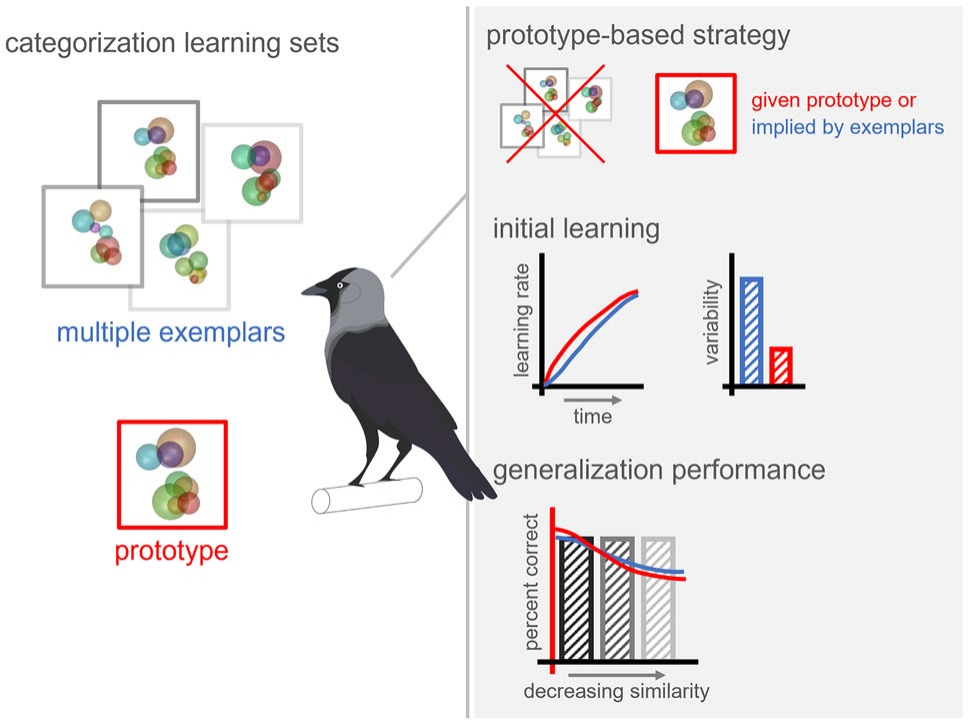

**Supplementary Information:**

The online version contains supplementary material available at 10.1007/s00429-023-02651-w.

## Introduction

After a long day, you decide to have an after-work drink with your colleague. Your favorite café serves a wide variety of wine so, knowing your all-time favorite, you order a ‘Primitivo’. Your colleague just lists all wine varieties he liked at the last wine tasting instead of ordering something specific. Unfortunately, the bartender informs you that ‘Primitivo’ is sold out and that none of the wines from the tasting are part of the regular menu. However, he assures you that he will find the perfect wine for both of you. Yet, how can he solve this task efficiently? The similarity to the specific example (i.e., a good alternative for the ‘Primitivo’) or to common features of the full list of wines could be informative for a correct choice. Beyond the selection of fitting wines, these two different approaches represent two fundamental properties of categorization, a crucial element of cognition relevant to survival in a constantly changing environment (Mervis and Rosch 1981; Herrnstein 1990; Smith et al. 2016; Lazareva and Wasserman 2017). Through categorization, an animal reduces the complexity of a stimulus to a set of features sufficient for adaptive behavior (Jitsumori and Delius 2001; Smith et al. 2016). Thereby, categorization optimizes the use of working memory for information processing (Panichello et al. 2019).

When confronted with a stimulus comprising all diagnostic features (i.e., the category prototype), subsequent stimuli can be directly compared to this ideal example (Posner and Keele 1968; Reed 1972; Jitsumori and Delius 2001; Minda and Smith 2001; Smith et al. 2016). Relative similarity to this prototype defines whether other stimuli are assigned into the same category or not, based on gradual category borders (Harnad 1987; Bowman et al. 2020). Within this framework, a category contains members that differ in their representativeness. Category members with diverging similarity can be learned, which results in a continuous expansion of the perceived category (Mervis and Pani 1980). Thus, an initially narrow category (with high prototype similarity) is transformed into a broader category that includes a wider range of members diverging from the prototype in non-diagnostic features.

Yet, some situations require a differentiation of individual category members, favoring category representations that retain stimulus-specific information (e.g., differentiating predator types (Seyfarth et al. 1980)). Such exemplar-based categorization requires memorization of individual stimuli and can therefore successfully represent perceptually incoherent or highly abstract categories (e.g., oddball exceptions (Castro et al. 2021)). Early category learning can be based on multiple exemplars, i.e., a diverse set of stimuli that span a large range of category features (“multiple cognitive reference points” (Medin and Schaffer 1978; Smith et al. 2016, p. 3; Bowman et al. 2020)). In the absence of a category prototype, new stimuli must be compared to previously seen exemplars (Medin and Schaffer 1978; Homa et al. 1981; Nosofsky 1986, 1987; Bowman et al. 2020). Thus, successful categorization depends on stimulus familiarity and initial categorization is difficult, because higher variability interferes with learning (Raviv et al. 2022). This process requires higher memory demands (due to memorization of all individual exemplars (Nosofsky 1987)) but results in a broader, more stable category representation (Hahn et al. 2005; Nosofsky et al. 2019; Raviv et al. 2022).

To process categories efficiently, it might be advantageous to construct a central category prototype from individual exemplars (Homa et al. 1981). This requires the identification of general diagnostic features of a stimulus set (Kruschke 1992; Sigala et al. 2002; Cook and Smith 2006) and facilitates categorization of stimuli that share a certain perceptual similarity (“prototype representations […] as a byproduct of retrieving category exemplars” (Bowman et al. 2020), p. 3). Humans excel at this ability (Tiedemann et al. 2022), and beyond primates, birds offer a unique perspective to compare evolutionary trends of categorization. Targeting different aspects of categorization has revealed birds’ cognitive aptitude regarding perceptual, rule based, and abstract categories (Aust and Huber 2002; Cook and Smith 2006; Katz and Wright 2006; Ditz and Nieder 2016; Peissig et al. 2019; Zipple et al. 2019; Anderson et al. 2020; Vernouillet et al. 2021). In particular, corvid songbirds can solve a large variety of cognitive tasks as successfully as primates (Güntürkün and Bugnyar 2016), and are able to perform categorization of highly abstract and complex stimuli (Veit and Nieder 2013; Ditz and Nieder 2015; Wagener and Nieder 2020). For instance, several corvid species successfully learned the concept of same/different (Vernouillet et al. 2021), with crows even flexibly alternating according to specific behavioral rules (‘match/nonmatch rule’, (Veit and Nieder 2013)). Further, the ability of crows to distinguish visual stimuli solely based on numerosity was extensively studied, revealing successful categorization on a high abstraction level and spontaneous categorical representations of numerosity on the neuronal level (Wagener et al. 2018). Even beyond the visual domain, crows were shown to master auditory categorization (Wagener and Nieder 2020). Yet, most studies so far focused on the categorization behavior following prolonged behavioral training and not on categorization learning itself.

The processing of visual information in birds involves various hierarchically organized brain regions, suggesting that birds may have different types of category representations, ranging from low-level perceptual stimulus features in primary sensory areas, to more abstract categorical representations in higher associative areas (Soto and Wasserman 2014; Clark and Colombo 2020; Pusch et al. 2022). However, it remains unclear how previous experience shapes the formation of distinct category representations, and to what degree specific forms of categorization depend on the behavioral task and the category structure used during learning (i.e., single stimulus vs. diverse stimulus pool). Given the adaptive behavior of corvids to a wide range of cognitive challenges, would they construct a category prototype to efficiently categorize a subset of similar exemplar stimuli? Furthermore, does the construction of an intrinsic prototype representation require as few trials as for humans (Xu and Tenenbaum 2007; Tiedemann et al. 2022) and computer simulations (Smith 2014)?

To resolve these questions, we trained jackdaws (*Corvus monedula*) to learn to categorize a large number of novel stimuli within single experimental sessions. The birds performed two variations of a delayed match to category task in which a category prototype was either directly presented (‘prototype approach’) or could be constructed from a large number of diverse category members (‘exemplar approach’). We hypothesized that the jackdaws would predominantly use a prototype-based strategy, even if the way they experienced the stimuli was based on a diverse set of category exemplars. Our results suggest that indeed a focus on a general category representation, instead of individual stimuli, is the predominant mechanism by which the animals learn to make categorical judgements.

## Methods

### Subjects

This experiment was performed with two experimentally naïve jackdaws (*Corvus monedula*) of undetermined sex (4 years of age), that were housed in a large indoor aviary in a social group (approximately 20 to 22 °C room temperature, 12-h day–night circle, including 30-min twilight phases, artificial daylight conditions with UV light, full color spectra, and high frequent illumination (5 kHz), ME International, Gallux). Both water and grid were available ad libitum and a controlled food protocol was used during the experiment (both birds were trained above 85% of their free feeding weight at 190 and 220 g). The birds obtained special bird food pellets as reward during training (NutriBird F16, Versele Laga) and a mix out of seeds, dried/fresh fruits, dried insects, mealworm larvae, and two bird foods (Beo-Weichfutter, Trocken-Weichfutter III braun, Claus) on days without training, supplemented with Korvimin (vitamin product, ZVT + Reptil). All experimental conduct was in agreement with the European Communities Council Directive for the care and use of animals for experimental purposes and approved by the local authorities (LANUV NRW).

### Apparatus

A darkened operant conditioning chamber (80 cm × 54 cm × 56 cm (height x width x depth)) served for training and testing. An acoustic pulse touchscreen (22’’, ELO 2200 L APR, Elo Touch Solutions Inc., CA) was used for stimulus presentation and to register peck responses. Food reward was delivered via an automated pellet feeder (https://www.ngl.psy.ruhr-uni-bochum.de/ngl/shareware/pellet-feeder.html.en). The birds were seated on a wooden perch (distance to monitor approximately 10.5 cm). A computer running custom MATLAB code using the Psychophysics (Brainard 1997) and Biopsychology toolboxes, OTBR (Rose et al. 2008), controlled all experimental procedures.

### Stimulus generation

**Fig. 1:**
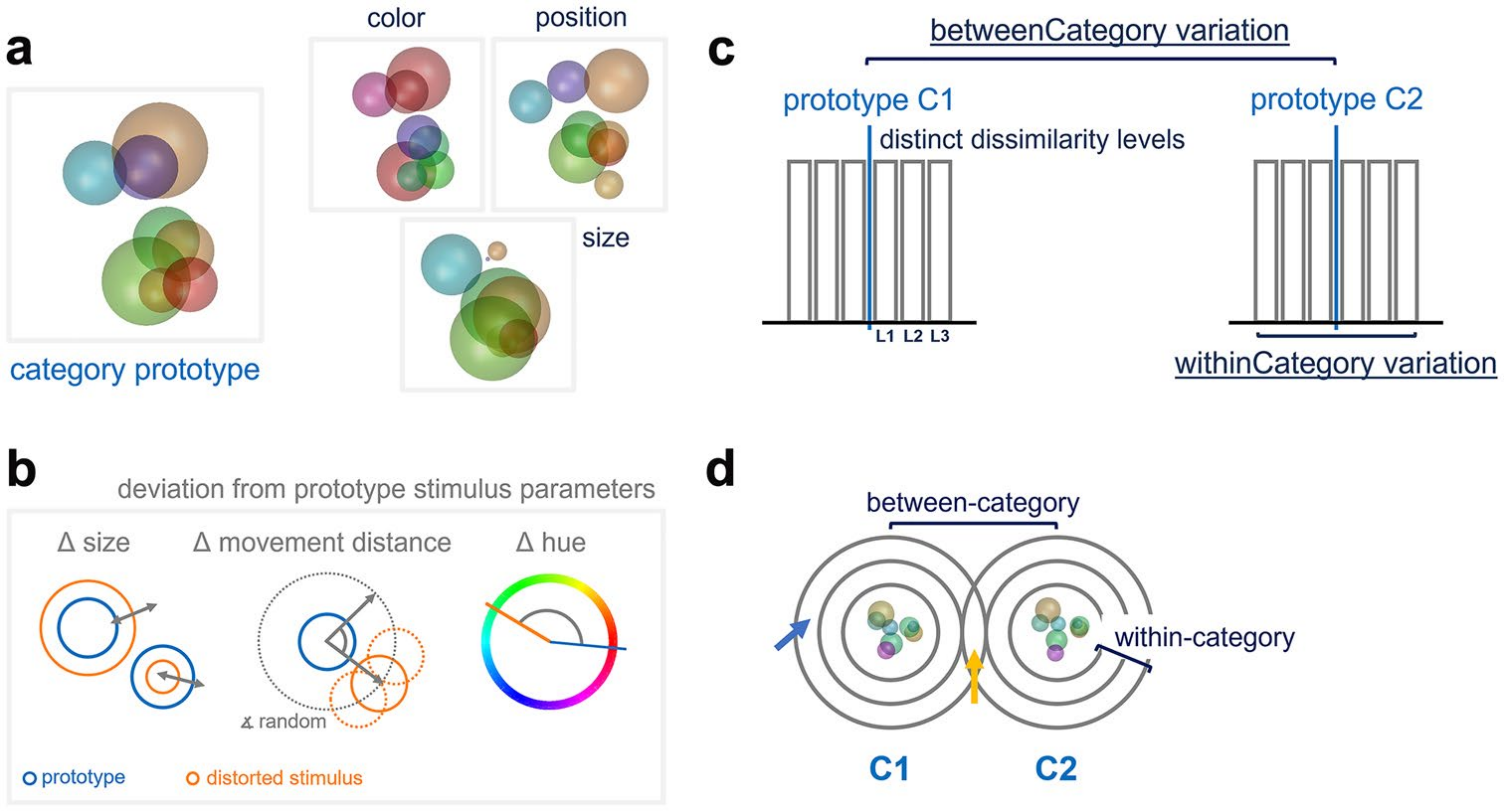
RUBubble stimuli were used to create categorization stimulus sets. **a** Three different stimulus parameters (sphere size, color, and position) could be manipulated separately. **b** Each category was created based on one randomly generated category ‘prototype’, from which individual stimuli were derived (‘distorted stimulus’, visualized in orange). The number of spheres in all stimuli was fixed to eight. Minimum and maximum deviations per stimulus parameter (size, movement distance, and hue) were specified relative to the category prototype when creating new category members. **c** Within- and between-category variation could be controlled. Stimuli used in one session were always created based on two distinct category prototypes that were related to each other. Prototype ‘C2’ was derived from prototype ‘C1’, which allowed precise control of between-category variation by setting the exact deviation of all stimulus parameters between the two stimuli. Within-category variation was specified by the maximum deviations of all stimulus parameters relative to their respective category prototype. **d** Schematic illustration of two categories created from two prototypes depicted in the center. Circles visualize associated category stimuli in distinct dissimilarity levels relative to their prototype in a simplified way. When creating members of a given category, specified values defined the deviation from the central category prototype without any directionality. Thus, the resulting category can be visualized as a distribution around a centered category prototype. Depending on the random direction of variation, each individual sphere of some category stimuli (in particular such of higher within-category dissimilarity) could be either more similar (yellow arrow), or less similar (blue arrow) relative to the opposing category protoype with regard to individual stimulus parameters (generally possible to have some overlap between two categories but unlikely concerning the whole stimulus). Category stimuli were subdivided into distinct similarity levels based on their parameter deviations relative to the corresponding prototype. This simplification was used to reduce the complexity within the stimulus set (independent modification of position, size, and color of each individual sphere within each individual stimulus) and turn it into a more accessible similarity level. RUBubble stimuli in **a** and depiction in **b** modified from *(*Apostel and Rose 2021*)*.

We used a novel, highly flexible artificial categorization stimulus type, ‘RUBubbles’ (Apostel and Rose 2021). Each generated RUBubble stimulus consisted of eight colored spheres that were arranged in 3D but shown as 2D images (Fig. 1a). Each stimulus category was generated based on one central, randomly created category prototype, for which the only fixed input was the number of desired spheres. Within-category similarity was specified by setting the minimum and maximum deviation per stimulus parameter (separately for hue, position, and size of spheres, Fig. 1b, c). We further subdivided these parameter ranges to create category members belonging to six distinct dissimilarity levels relative to the category prototype (category prototype = L0, category members L1–L6). Deviations per stimulus parameter specified the respective distance relative to the corresponding prototype. Thus, individual dissimilarity levels can schematically be envisioned as distributions around the centered prototype, with varying distance (spatially with x, y, and z; color and size along a line) from the corresponding prototype spheres. Stimulus parameters could differ in their similarity relative to the other category prototype (see arrows, Fig. 1d), which might result in some overlap between both category distributions regarding individual features (however, unlikely to concern all stimulus dimensions simultaneously). Overall, we created novel sets for each experimental session containing 481 category members (1 prototype and 80 stimuli per dissimilarity level for each category). To control between-category similarity, the prototype of category 2 was derived from the prototype of category 1. For this, we specified the exact deviation of position (as movement distance between corresponding spheres), color, and size between both prototypes (Fig. 1c). Categorization stimulus sets were individualized for each bird to adjust the difficulty level of the task. Thus, the minimum and maximum deviation ranges differed between birds and sessions.

### Behavioral task

The birds initiated each trial by responding to a white dot. After a 500 ms delay, a sample was presented for 1 s that had to be memorized across a delay period of 1 s. After the delay, two stimuli were presented as choice and the birds had to select the stimulus matching the category of the previously presented sample (delayed match to category, A/B categorization task (Zeithamova et al. 2008), Fig. 2a). Correct responses were rewarded with food pellets, whereas incorrect responses were signaled via a brief screen flash followed by a short time out.

**Fig. 2:**
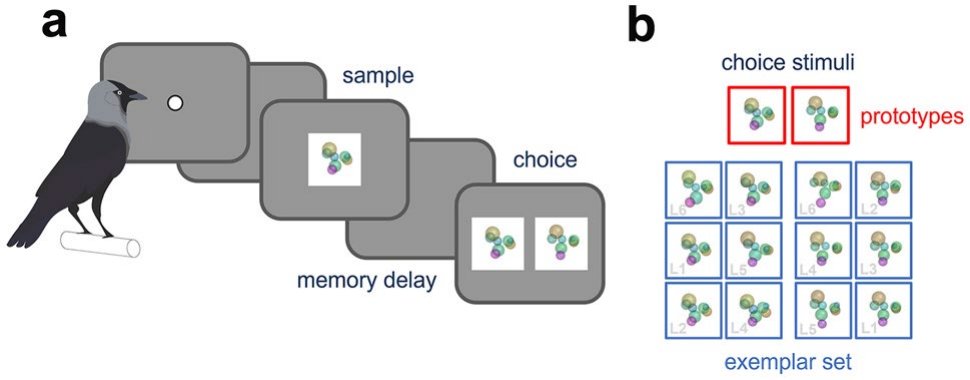
Schematic illustration of the delayed match to category paradigm and both training approaches. **a** After initiating a trial by pecking a white dot, a sample was presented for 1 s, which was followed by a 1-s delay period. During choice, the birds had to select the stimulus representing the matching category. Correct responses were rewarded, and incorrect responses were signaled by a screen flash followed by a short time-out period before the next trial was presented. **b** Two different variations of the delayed match to category paradigm were implemented. ‘Prototype’ and ‘exemplar’ sessions differed in the selection of sample stimuli and the composition of the choice stimulus array. In ‘prototype’ sessions, sample stimuli with increasing dissimilarity level were presented sequentially, making the initial variability low. In ‘exemplar’ sessions, all levels of dissimilarity were included from the beginning, increasing the initial variability. The birds had to make their response by pecking either the matching category prototype (L0, ‘prototype’ sessions, red) or an exemplar stimulus matching the sample category (subset of all category stimuli as choice stimulus pool in ‘exemplar’ sessions, L1–L6, blue)

Two variations of a delayed match to category paradigm were used to differentiate distinct forms of categorization learning: a prototype- and an exemplar-based approach. We aimed to specifically manipulate the underlying stimulus set variability, showing either one single stimulus (i.e., the category prototype) or a diverse set of various stimuli (i.e., the subset of category exemplars). In our study, these two training protocol variations defined the type of session, i.e., ‘prototype session’, and ‘exemplar session’. Individual trials were structured identically in both variants, as described above. However, the selection of presented sample and choice stimuli was dependent on the session type (Fig. 2b).

In ‘prototype’ sessions, the birds were presented with two category prototypes in the very first trial. Each explicit category prototype was presented as both sample and choice stimulus (Fig. 2b, red). Only as the session progressed, other category members were introduced as sample stimuli, being progressively less similar to the prototypes (slowly increasing variability). Thus, what effectively began as a delayed match to sample task (i.e., select the choice stimulus identical to the sample) was transformed into a delayed match to category task (i.e., select the choice stimulus belonging to the same category as the sample). The category prototypes were kept as the choice stimuli throughout the entire ‘prototype’ session. Thus, the birds always selected the prototype belonging to the presented sample category when responding.

In ‘exemplar’ sessions, all stimuli per trial (one sample and both choice stimuli) were randomly selected from the full RUBubble category. The birds experienced stimuli with varying dissimilarity to the category prototypes from the beginning and thus exclusively performed a delayed match to category task. Consequently, the category prototypes were not explicitly introduced, in contrast to the ‘prototype’ sessions. Throughout the ‘exemplar’ session, a subset of category stimuli spanning the full category was used as choice stimuli (Fig. 2b, blue).

### Block design

**Fig. 3:**
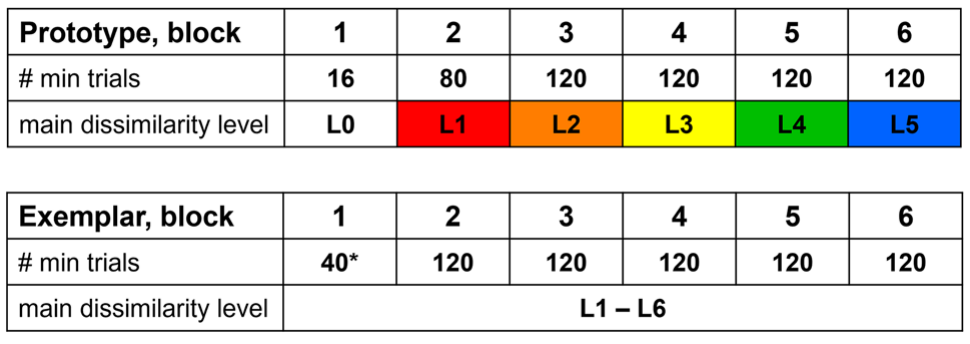
Overview of block design implemented in 'prototype' (top) and 'exemplar' sessions (bottom). Prototype: block 1 in ‘prototype’ sessions resembled a delay match to sample task with only category prototypes (L0) used both as sample and choice stimuli. Main dissimilarity level increased across blocks (L0–L5, color coded). Exemplar: sample stimuli were randomly chosen from all six dissimilarity levels in each block; thus, ‘exemplar’ sessions showed no consistent relation between block number and dissimilarity level. *In some ‘exemplar’ sessions, block 1 contained a minimum of 80 instead of 40 trials (first 28 (bird 1), and 22 (bird 2) ‘exemplar’ sessions, later reduced to increase the number of completed trials in full blocks for analysis). All full blocks contained a minimum of 120 trials in both session types.

Each session was divided into six potential blocks. The animal’s performance during a block decided when a block was completed, and two different criteria had to be met to pass over into the next block. First, specific trial conditions had to be used in at least 20 completed trials, to ensure a sufficient number of trials (conditions explained in the next section). Second, the birds had to respond with at least 80% correct choices within the last 20 completed trials (performance criterion). Thus, the number of blocks per session and the number of trials per block were dependent on the behavioral performance. In ‘prototype’ sessions, each block was associated with one main dissimilarity level (L, color coded in Fig. 3). Throughout the session, sample stimuli became progressively more dissimilar to the prototype as the main dissimilarity level of sample stimuli per block increased. Block 1 involved only the category prototypes (L0), both as sample and choice stimuli (i.e., initial task consistent with delayed match to sample paradigm). In ‘exemplar’ sessions, each block contained sample stimuli from all six dissimilarity levels. A detailed description of trial conditions and block design will be given in the following sections.

### Trial conditions (sample familiarity)

In addition to the six distinct dissimilarity levels relative to their prototype, sample stimuli were distinguished based on their familiarity into:

*Familiar* (*F*)—stimuli that had been presented in a preceding block. In ‘prototype’ sessions, F stimuli always had a dissimilarity level in accordance with the current block (i.e., the main dissimilarity level).

*Novel* (*N*)—novel stimuli. In ‘prototype’ sessions, novel stimuli were additionally differentiated based on their respective dissimilarity level. Novel stimuli with a dissimilarity level consistent with the current block were indicated as NF (i.e., novel stimuli of a familiar dissimilarity level). Novel stimuli with a dissimilarity one level higher than the current block were indicated as N (i.e., novel stimuli of a novel dissimilarity level). In ‘exemplar’ sessions, N stimuli were taken randomly from all dissimilarity levels.

**Fig. 4:**
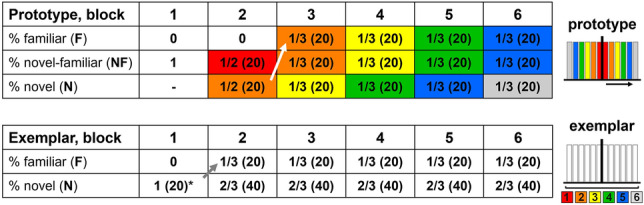
Detailed overview of block design and trial conditions (i.e., sample familiarity), in ‘prototype’ and ‘exemplar’ sessions. Prototype: block 1 in ‘prototype’ sessions involved only the category prototypes (L0). Block 2 included sample stimuli from dissimilarity level L1 and L2 (only novel). Block 3–6 represented full blocks, containing 20 familiar sample stimuli that had been introduced as novel samples in the previous block respectively (white arrow). Novel sample stimuli belonged either to the same dissimilarity level as familiar samples (e.g., NF from L2 in block 3, orange) or to the next dissimilarity level (e.g., N from L3 in block 3, yellow). This pattern was maintained in each subsequent block. Thus, sample stimuli from specific dissimilarity levels only appeared in two subsequent blocks (with the exception of L1 and L6). For bars on the right, dissimilarity levels are color coded, and the centered black line represents the category prototype. Exemplar: block 1 contained 20 novel stimuli that reappeared as familiar stimuli in block 2 (grey arrow). In the early ‘exemplar’ sessions (first 28 for bird 1, and first 22 for bird 2), block 1 contained 40 novel stimuli per category (noted as ‘*’). This was later reduced to 20, to increase the number of trials in later blocks. Each full block consisted of 20 familiar and 40 novel sample stimuli selected from all dissimilarity level (F samples in any given block were N samples in the preceding block)

Individual sample stimuli were used at most in two consecutive blocks (e.g., N stimuli in block 3 reappeared as F samples in block 4). Figure 4 gives a schematic overview of sample details.

In ‘prototype’ sessions, block 2 contained only novel stimuli from two different dissimilarity levels (20 NF (L1) and 20 N (L2) per category, for a total of 80 stimuli), as the only familiar stimuli would have been the prototypes (Fig. 4). Each subsequent block then always included 20 F, 20 NF, and 20 N stimuli per category (for a total of 120 stimuli). Overall, each block was associated with one main dissimilarity level (Fig. 3 top). For instance, the majority of sample stimuli in block 3 belonged to dissimilarity level 2 (L2, highlighted in orange, Fig. 4). In ‘exemplar’ sessions there was no consistent relation between block and dissimilarity level since each block contained stimuli from all dissimilarity levels (Fig. 3 bottom). Thus, sample stimuli were only distinguished as familiar or novel (Fig. 4 bottom). Block 1 contained only 20 N stimuli per category (for a total of 40 stimuli), which introduced the categories without showing an explicit prototype. All subsequent blocks then contained 20 F and 40 N sample stimuli per category (for a total of 120 stimuli, Fig. 4 bottom).

### Randomization

The behavioral task dynamics needed to be flexible to fulfill all requirements of performance-dependent parameters that could not be predefined. To that end, specific trial conditions labeled according to block (1–6), sample category (C1, C2), and sample type (F, NF, N) were predefined and randomized within each block before the session started. An extensive number of conditions per block were generated, which was necessary, because the actual required number of trials was dependent on the behavioral performance. Individual sample stimuli were assigned and listed online during the ongoing behavioral task. This enabled us to present N stimuli as F stimuli in the next block (by, e.g., ignoring trial omissions). Whenever the required 20 trials per sample type had been presented, but the performance criterion had not yet been met, we repeated previously presented sample stimuli within the block. These ‘repetition’ trials were excluded from later familiarity analyses.

### Data collection and statistical analysis

Custom code written in MATLAB (Mathworks, R2018b, R2020b) using the Psychophysics (Brainard 1997) and OTBR toolboxes (Rose et al. 2008) was used to run the experimental paradigm and to save and analyze the behavioral data. All completed trials (apart from repetition trials for familiarity analysis) were analyzed.

To investigate differences between session types with respect to initial learning of categories, we applied a *χ*^2^-test on the obtained learning curve slopes within the first 100 trials, after removing the trials of the first block of ‘prototype’ sessions, as those represented a delayed match to sample instead of a categorization task. To do so, we binned the binary trial-by-trial categorization of a stimulus (1—correct; 0—false) across five consecutive trials (moving with a sliding window across the first 100 trials, at a step width of one). We counted the total amount of correct and false per bin, per session, per session type, and thereof calculated the *χ*^2^ statistic (Table S2). We considered slopes as significantly different between ‘prototype’ and ‘exemplar’ sessions if two consecutive, non-overlapping bins were individually significant at an alpha of 0.05.

We further investigated the effect of different independent variables (i.e., session type, block, proportion of familiarity, dissimilarity, Δ-level, and familiarity) on behavioral performance as the main dependent variable. Performance was measured as percent correct within blocks per session and for specific trials (e.g., the first 100 trials of each session) between sessions. Both of these measures were sampled between sessions.

To detect an effect of category experience, we performed a two-way ANOVA with factors session type (i.e., ‘prototype’ or ‘exemplar’), block (1–5), and the interaction between these factors. We further analyzed the dependency of performance on overall similarity in ‘exemplar’ sessions by performing a one-way ANOVA with factor proportion of familiarity. To investigate effects of distinct stimulus features, we performed a separate one-way ANOVA for the factor dissimilarity (between sample and respective category prototype), for both ‘prototype’ (five levels) and ‘exemplar’ sessions (six levels), and a one-way ANOVA for the factor Δ-level (dissimilarity level deviation between sample and matching choice stimulus in ‘exemplar’ sessions, five levels). The effect of sample familiarity in ‘prototype’ sessions was investigated by one-way ANOVAs with the factor familiarity (pooled per block (i.e., F, N, and NF of the same level, test between blocks), and dissimilarity levels (i.e., F, N, and NF of the same block, test between dissimilarity levels)). In ‘exemplar’ sessions, effects of stimulus familiarity were investigated by dependent t tests. We pooled stimuli according to dissimilarity level across the entire session (i.e., testing F vs. N stimuli per dissimilarity level, irrespective of block) and across block 2 (i.e., testing F vs. N within block 2, for each dissimilarity level).

As an effect size measure, we report $\omega^{2}$ (main factor of one-way ANOVAs) and $\omega_{p}^{2}$ (partial $\omega^{2}$, for main factors and interaction in two-way ANOVAs), which we interpret as the percentage of explained variance (PEV) by the respective factor. Any statement about significance following from post hoc comparisons between levels of an ANOVA used a corrected alpha level, applying Tukey’s honestly significant difference procedure in MATLAB. Other statements of significance were made based on appropriate corrections of the alpha level using the Bonferroni method.

We conducted Bayesian statistics for all t tests and ANOVAs using the open-source data analysis software JASP (JASP Team 2023). Support for null models (i.e., assuming no factor driven differences) and for factor driven models is reported for each individual analysis. To quantify support, we report Bayesian factors for either the null model (BF_null_), or the factor driven model (BF_M_), depending on which model had higher support given the observed data. All tested models were given equal prior probability, the resulting posterior probability given the observed data (P(model|data)) is reported for the more probable model. For analyses with only two competing models the posterior probability of the less probable model can thus be inferred.

## Results

### Birds categorized session-unique stimuli demonstrating fast initial learning due to explicit category prototypes

Our jackdaws gained extensive experience in categorizing arbitrary RUBubble stimuli under two distinct learning approaches. In total, bird 1 and bird 2 performed 24,317 and 28,960 prototype trials (an average of 458.81 (± 45.92) and 490.85 (± 51.18) trials per session; across 53 and 59 sessions, respectively), and 28,960 and 26,750 exemplar trials (an average of 477.85 (± 35.21) and 514.42 (± 39.68); across 60 and 52 sessions, respectively). Each session contained two session-unique sets of RUBubble stimuli belonging to categories C1 and C2. In ‘prototype’ sessions, the birds initially experienced each category’s de facto prototype and encountered members of either category with gradually reduced similarity from their respective prototype. In ‘exemplar’ sessions, the birds were confronted with stimuli representing the full range of possible similarities within their respective category but never encountered the actual prototype.

The number of blocks per session was dependent on the behavioral performance with a given stimulus set and, to some extent, reflected the respective stimulus set difficulty. Overall, both birds reached the higher blocks (i.e., ≥ 4) in most experimental sessions (Fig. S1a, b, for comparability, our analysis included only sessions in which the birds reached at least block 4). In ‘prototype’ sessions, the birds generally correctly categorized sample stimuli from dissimilarity levels up to L4 (i.e., block 4/5, 88.33% and 88.06% of all ‘prototype’ sessions for birds 1 and 2, respectively). Similarly, in ‘exemplar’ sessions, bird 1 most commonly reached block 4 or 5 (73.17% of sessions), and bird 2 most commonly reached block 3 or 4 (73.68% of sessions; note that in ‘exemplar’ sessions, all blocks contained stimuli from all six dissimilarity levels, i.e., up to L6). Both birds successfully categorized unfamiliar RUBubble category sets in individual sessions (indicated by reaching the higher blocks) with either prototype- or exemplar-based approach without showing a chronological dependence on session (Fig. S1 c & d).

**Fig. 5:**
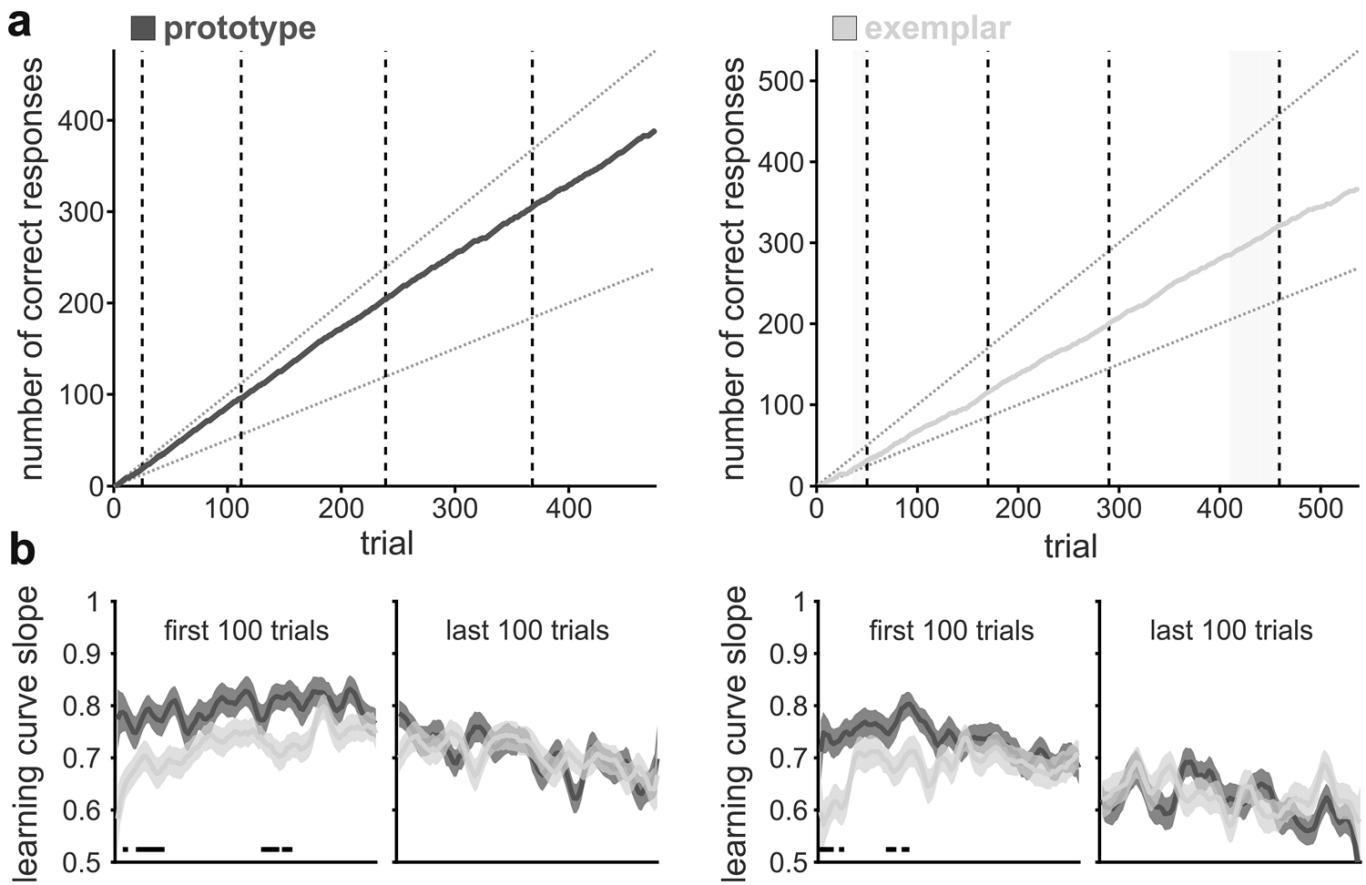
Performance increase was steeper in ‘prototype’ sessions. **a** Cumulative number of correct trials throughout exemplary ‘prototype’ (bird 1, left) and ‘exemplar’ (bird 2, right) sessions. In ‘prototype’ sessions, the initial performance was generally higher in comparison with ‘exemplar’ sessions. Diagonal dotted lines represent perfect and chance performance (100% vs. 50% correct). Transitions between distinct blocks are indicated by the vertical dashed lines. Grey shading indicates trials in which the required number of trials per condition was reached; however, performance within the last 20 trials still below 80% correct. The final block reached was the 5^th^ in both example sessions. **b** General overview of learning curve slopes for each bird within the first and last 100 trials of ‘prototype’ (dark grey) and ‘exemplar’ (light grey) sessions. The average slope in ‘exemplar’ sessions was below that of ‘prototype’ sessions for both birds within the first 100 trials, indicating a slower initial learning. This difference disappeared towards the end with similar learning curve slopes throughout the last 100 trials in ‘prototype’ and ‘exemplar’ sessions. Perfect performance would be reflected in a learning curve slope of 1, chance performance in a slope of 0.5. Slope was calculated as first derivative of each learning curve using a sliding window of 5 trials, and then averaged across sessions and visualized as mean ± SEM (block 1 of ‘prototype’ sessions was excluded from this analysis as it represented no true categorization task). Black bars at the bottom indicate significant differences, based on a *χ*^2^-test. Bird 1 left, *n* = 53 and 60 sessions; bird 2 right, *n* = 59 and 52 sessions (‘prototype’ and ‘exemplar’, respectively)

We tracked the birds’ performances in individual sessions with learning curves showing the cumulative number of correct responses (Fig. 5a). Learning curve slope was generally smaller (closer to chance) in the earlier blocks of ‘exemplar’ sessions (Fig. 5a, right), in comparison with initially very high performance in ‘prototype’ sessions (Fig. 5a, left). Across all sessions, the average learning curve slope was slightly higher in ‘prototype’ than ‘exemplar’ sessions throughout the first 100 trials (Fig. 5b, significant differences in learning curve slopes between ‘prototype’ and ‘exemplar’ sessions indicated by black bars, bird 1: all $\chi_{\left( 1 \right)}^{2} \,\, \ge \,\,4.4291, \,all \,p\,\, \le \,\,0.0353,$ bird 2: all $\chi_{\left( 1 \right)}^{2} \,\, \ge \,\,4.2855,\, all \,p\,\, \le \,\,0.0384,$ see Table S3 for full results). At the end of both session types, this difference disappeared (average slope in last 100 trials per session, Fig. 5b, see supplementary Table S3 for full results). The overall decrease of learning curve slope towards the end in all sessions could have been due to a general decrease in motivation owing to, for instance, saturation (comparison of slope for the first vs. last 100 trials: $\chi_{\left( 1 \right)}^{2} = 113.22 , p < 0.0001,$ and$\chi_{\left( 1 \right)}^{2} = 189.37, \,p\,\, < \,\,0.0001,$, for birds 1 and 2, respectively).

### Behavior suggests prototype-based categorization over memorization of individual stimuli

**Fig. 6:**
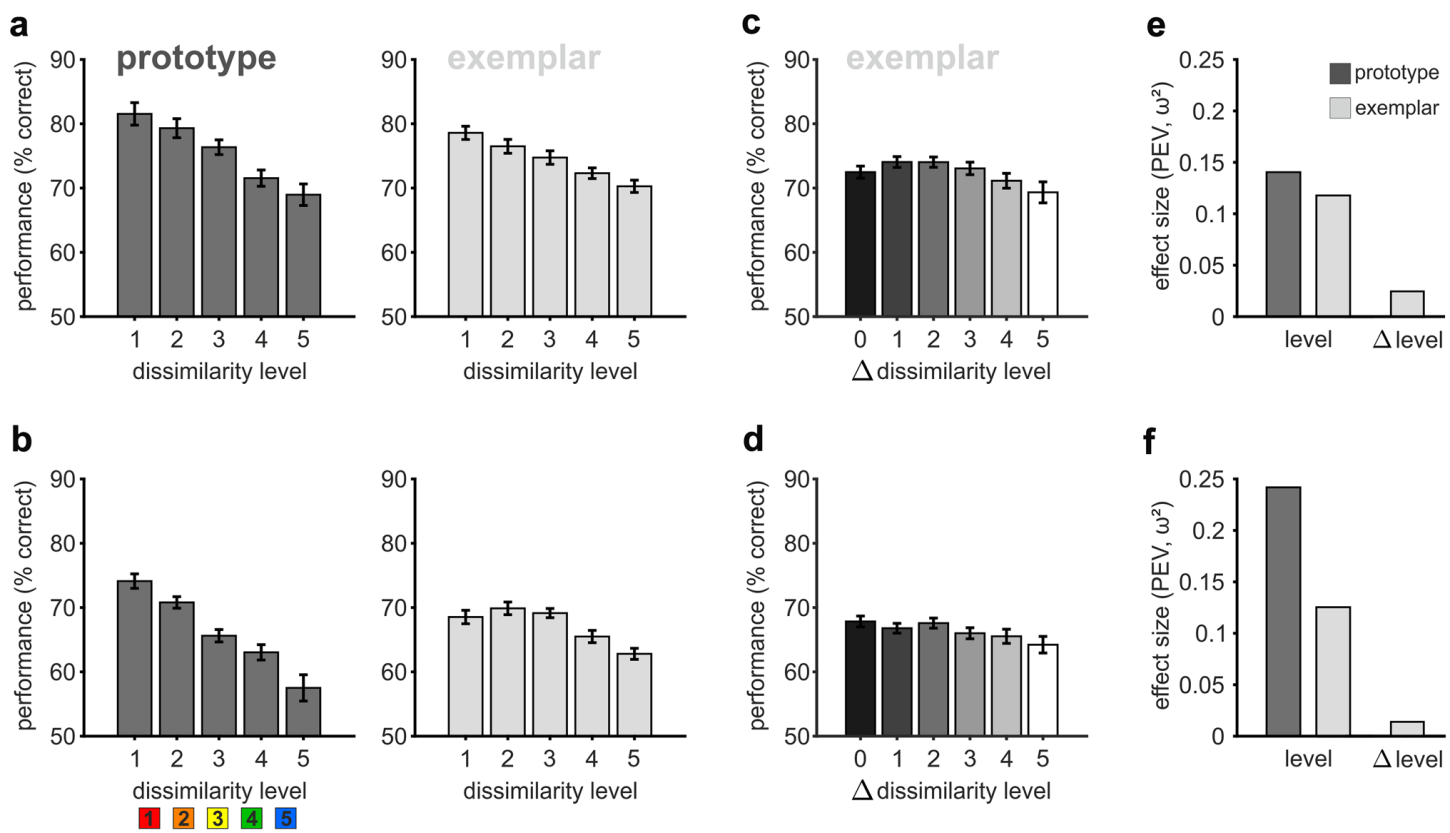
Categorization performance depended on sample dissimilarity level irrespective of the learning approach. **a**, **b** Categorization performance in ‘prototype’ sessions (dark grey) decreased with increasing sample dissimilarity level for both birds. Performance was calculated per session and dissimilarity level, and then averaged across all ‘prototype’ sessions. ‘Exemplar’ sessions (light grey) showed a similar dependency. The effect of dissimilarity level on performance was smaller but still present, even though no explicit prototype was shown. **c**, **d** Categorization performance in ‘exemplar’ sessions was mostly independent of the dissimilarity deviation between sample and matching choice stimulus (Δ-level). Performance was calculated per session and Δ-level per trial, and then averaged across all ‘exemplar’ sessions. **e**, **f** The similarity of match and choice stimuli in ‘exemplar’ sessions did not affect performance (Δ-level), whereas sample dissimilarity level influenced performance to a similar degree in ‘prototype’ and ‘exemplar’ sessions (level). Bird 1 top row, *n* = 53 and 60 sessions; bird 2 bottom row, *n* = 59 and 52 sessions (‘prototype’, and ‘exemplar’, respectively)

We aimed to characterize the different learning processes in the formation of novel categories based on disparate stimulus set variability with two distinct approaches, i.e., prototype-based and exemplar-based categorization. When evaluating a novel stimulus following a prototype strategy, the comparison to the central prototype is the main factor determining category membership (Bowman et al. 2020). Thus, categorization performance should be higher for stimuli that are more similar to the prototype. We visualized the performance per sample dissimilarity level in ‘prototype’ sessions and calculated an ANOVA with factor dissimilarity level to quantify the effect on categorization performance. Both birds showed a substantial decrease in performance with decreasing similarity between sample and corresponding prototype (Fig. 6a, b, left subplots, $F_{4,244} = 11.17, p\,\, < \,\,0.0001, \,\omega^{2} = \,0.1405,$ P(M|data) > 0.999, BF_M_ > 1000, and $F_{4,263} \,\, = \,\,22.38, \,\,p\,\, < \,\,0.0001,\,\,\omega^{2} = \, 0.2419,$ P(M|data) > 0.999, BF_M_ > 1000). It is important to note that dissimilarity level was confounded with trial number in ‘prototype’ sessions, since most dissimilar stimuli were only present towards the end of a session. Thus, the effect of general motivational state of the animal might have been part of the performance decline (see Fig. S2 and Table S1 for details). However, the effect of dissimilarity was already present for intermediate levels in the middle of the session, which indicates a strong effect that was unlikely to be driven solely by motivation.

**Fig. 7:**
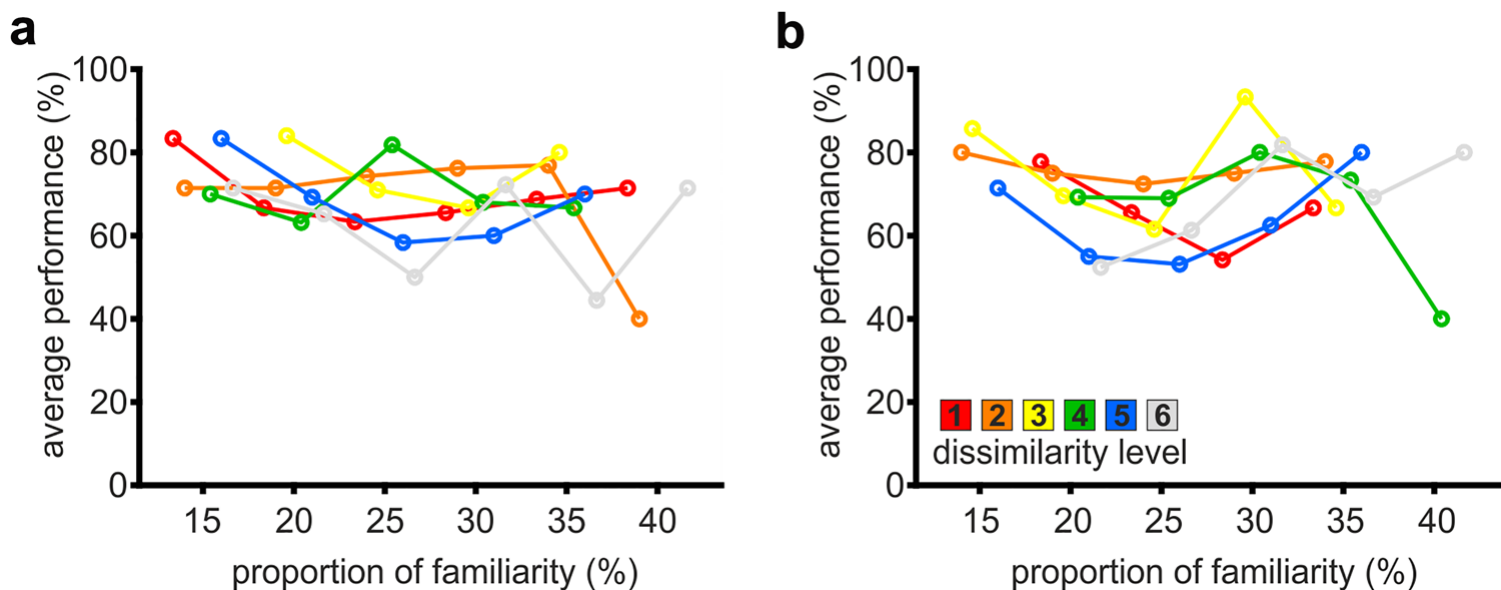
Categorization performance was independent of the overall similarity between sample and all previously presented stimuli. Overall similarity was calculated per trial including trials 11 to 30 of each ‘exemplar’ session. For example, in trial 11, the birds already encountered a total of 30 stimuli (10 sample and 20 choice stimuli). The occurrence of each dissimilarity level (L1–L6) was counted in all previous trials. For example, if counted quantities were 3, 5, 5, 6, 7, and 4 for L1, L2, L3, L4, L5, and L6, respectively, then L5 would have the highest proportion of familiarity (23.33%). If our birds were comparing the current sample to all previous stimuli, we would expect a higher performance for a sample belonging to L5 compared to one from L1, because the bird already encountered more stimuli from L5 (L5 sample with higher overall similarity relative to all previously encountered exemplars). Performance was averaged across all trials featuring the same proportion of familiarity for a given dissimilarity level and binned in steps of 5 percentage points, resulting in six discrete values. We used this approach as surrogate to estimate the effect of known exemplars. We found that the average performance of both birds was independent of the overall similarity of each dissimilarity level. See Fig. S3 for a detailed visualization including SEM. **a** bird 1, *n* = 60 sessions; **b** bird 2, *n* = 52 sessions

Learning to categorize from multiple exemplars involves memorization of individual stimuli (Medin and Schaffer 1978; Nosofsky 1986). In an exemplar-based strategy, novel sample stimuli are compared to all previously encountered and memorized exemplars of each category instead of a central prototype. Thus, performance should be better for stimuli that are more similar to already encountered exemplars. To investigate this, we analyzed performance based on the similarity of a given sample to all previously seen stimuli (focusing on trials 11–30). To obtain an estimate of the overall similarity between sample and already presented stimuli, we counted how many times the dissimilarity level of the current sample was already used either as sample or choice stimulus. From this, we calculated the proportion of familiarity per level as approximation of overall similarity (by dividing counts through total number of stimuli used). Then, we computed the mean performance as a function of the estimated proportion of familiarity across all ‘exemplar’ sessions for trials with the same sample dissimilarity level and proportion of familiarity. We hypothesized that, if birds were relying on exemplars, the performance should increase with higher proportions of already encountered, similar stimuli. However, we found that the proportion of previous stimuli with the same dissimilarity level as the current sample did not affect performance (one-way ANOVA of proportions of familiarity, $F_{5,26} = 0.93,{ }p = 0.48,$ P(null|data) = 0.777, BF_M_ = 3.488 and $F_{5,23} = 1.45,{ }p = 0.24,$ P(null|data) = 0.657, BF_M_ = 1.917 Fig. 7).

Another indication for the use of memorized exemplars would be an improved performance for familiar, relative to novel stimuli. Previous studies demonstrated a positive effect of stimulus familiarity on exemplar-based categorization (Medin and Schaffer 1978). Thus, familiarity of a stimulus should have a beneficial effect in ‘exemplar’ sessions, but virtually no effect in ‘prototype’ sessions, which primarily rely on the similarity relative to the category prototype. We analyzed performance separately for sample familiarity in ‘prototype’ sessions (Fig. S4, one-way ANOVA with factor familiarity, all *F* ≤ 2.05, all *p* > 0.05, see Tab. S4 for detailed results) and ‘exemplar’ sessions (Fig. S5, dependent t test per dissimilarity level, all *t* ≤|1.37|, all *p* > 0.05, see Table S5 for detailed results). We found no significant difference between familiar and novel stimuli in either session type, for any bird, or any dissimilarity level (at Bonferroni-corrected alpha level). The birds instead showed a general, substantial effect of dissimilarity level (reduced performance due to higher dissimilarity) but no differences between familiar and novel stimuli. Even in the first full block of ‘exemplar’ sessions (i.e., block 2, containing only relatively few stimuli), the factor familiarity did not become significant (Fig. S6, dependent t test per dissimilarity level, all *t* <|1.61|, all *p* > 0.05, see Table S6 for detailed results). Thus, it appears that our birds did not use stimulus familiarity to categorize RUBubble stimuli in ‘exemplar’ sessions.

Overall, we found no evidence for a dependency of performance on the similarity to previously seen exemplars or their familiarity. This is in contrast to what would have been expected from an exemplar-based approach. As an alternative strategy, the birds could have made their categorization decision based on the similarity between sample and matching choice stimulus per trial (a common behavior in delayed match to sample tasks). Therefore, we analyzed if the similarity between sample and matching choice stimulus affected categorization performance, expecting a higher performance if both stimuli belonged to the same or adjacent dissimilarity levels. We calculated the performance per absolute dissimilarity level difference (Δ-level) between sample and match across all blocks in ‘exemplar’ sessions. Performance was significantly affected by the Δ-level between sample and match for bird 1 ($F_{5,354} = 2.81, \,p = 0.0167, \,\omega^{2} = 0.0245, \,P\left( {\text{M|data}} \right) = 0.434, \,BF_{M} = 0.766$) but not for bird 2 (${F_{5,306} = 1.91,}\, {p = 0.0932,} \,{\omega^{2} = 0.0143,} \,{P\left( {\text{null}|{\text{data}}} \right) = 0.844,} \,{BF_{{{\text{null}}}} = 5.410,}$ Fig. 6c, d). We further investigated if the effect of sample-to-choice dissimilarity might have been limited to the early blocks (1–3), by analyzing the performance per Δ-level in each block separately. However, in all blocks and across the entire session, the performance was quite similar for each absolute Δ-level deviation between sample and match, and there was no significant effect for any of the blocks (Table 1, Fig. S7).

**Table 1:** Overview of statistical results of separate one-way ANOVAs testing the effect of Δ-level per block in ‘exemplar’ sessions

Block	Bird 1	Bird 2
1	$F_{5,349} = 2.16, \,p = 0.0576, \,\omega^{2} = 0.0161,$	$F_{5,306} = 1.08, \,p = 0.3697, \,\omega^{2} = 0.0013$
	$P\left( {\text{null|data}} \right) = 0.838, \,BF_{{{\text{null}}}} = 5.181$	$P\left( {\text{null|data}} \right) = 0.960, \,BF_{{{\text{null}}}} = 23.805$
2	$F_{5,354} = 1.22, \,p = 0.2979, \,\omega^{2} = 0.0031$	$F_{5,306} = 2.93,\, p = 0.0134, \,\omega^{2} = 0.0300$
	$P\left( {\text{null|data}} \right) = 0.963, \,BF_{{{\text{null}}}} = 25.907$	$P\left( {\text{M|data}} \right) = 0.580, \,BF_{M} = 1.382$
3	$F_{5,354} = 1.50, \,p = 0.1892,\, \omega^{2} = 0.0069$	$F_{5,306} = 0.83, \,p = 0.5274, \,\omega^{2} = \, - 0.0027$
	$P\left( {\text{null|data}} \right) = 0.948, \,BF_{{{\text{null}}}} = 18.066$	$P\left( {\text{null|data}} \right) = 0.978,\, BF_{{{\text{null}}}} = \,\,43.838$
4	$F_{5,346} = 0.51, \,p = 0.7686,\, \omega^{2} = \, - 0.0070$	$F_{5,297} = 0.67, \,p = 0.6498, \,\omega^{2} = \, - \,0.0055$
	$P\left( {\text{null|data}} \right) = 0.990, \,BF_{{{\text{null}}}} = 98.405$	$P\left( {\text{null|data}} \right) = 0.979, \,BF_{{{\text{null}}}} = \,\,46.218$
5	$F_{5,145} = 0.14, \,p = 0.9817, \,\omega^{2} = \,\, - 0.0292$	$F_{5,69} = 0.28, \,p = 0.9216, \,\omega^{2} = \, - \,0.0503$
	$P\left( {\text{null|data}} \right) = 0.980, \,BF_{{{\text{null}}}} = \,\,49.285$	$P\left( {\text{null|data}} \right) = 0.941, \,BF_{{{\text{null}}}} = 16.016$

There was no significant effect for any block at a Bonferroni-corrected alpha level of 0.005. Bayesian statistics indicate good support for the null model (null|data), and only moderate support for the alternative model of Δ-level (M|data) in case of block 2 of bird 2

### Birds constructed an implicit prototype from a subset of category exemplars within the first few trials

Another possible strategy would be to construct an implicit prototype from the subset of exemplars already encountered. To investigate if our birds followed this approach, we analyzed their performance in ‘exemplar’ sessions as a function of sample dissimilarity level (following the ‘prototype’ session analysis from Fig. 6a, b). We further quantified the effect of dissimilarity level within all completed blocks in all ‘exemplar’ sessions performing a one-way ANOVA with factor level.

**Fig. 8:**
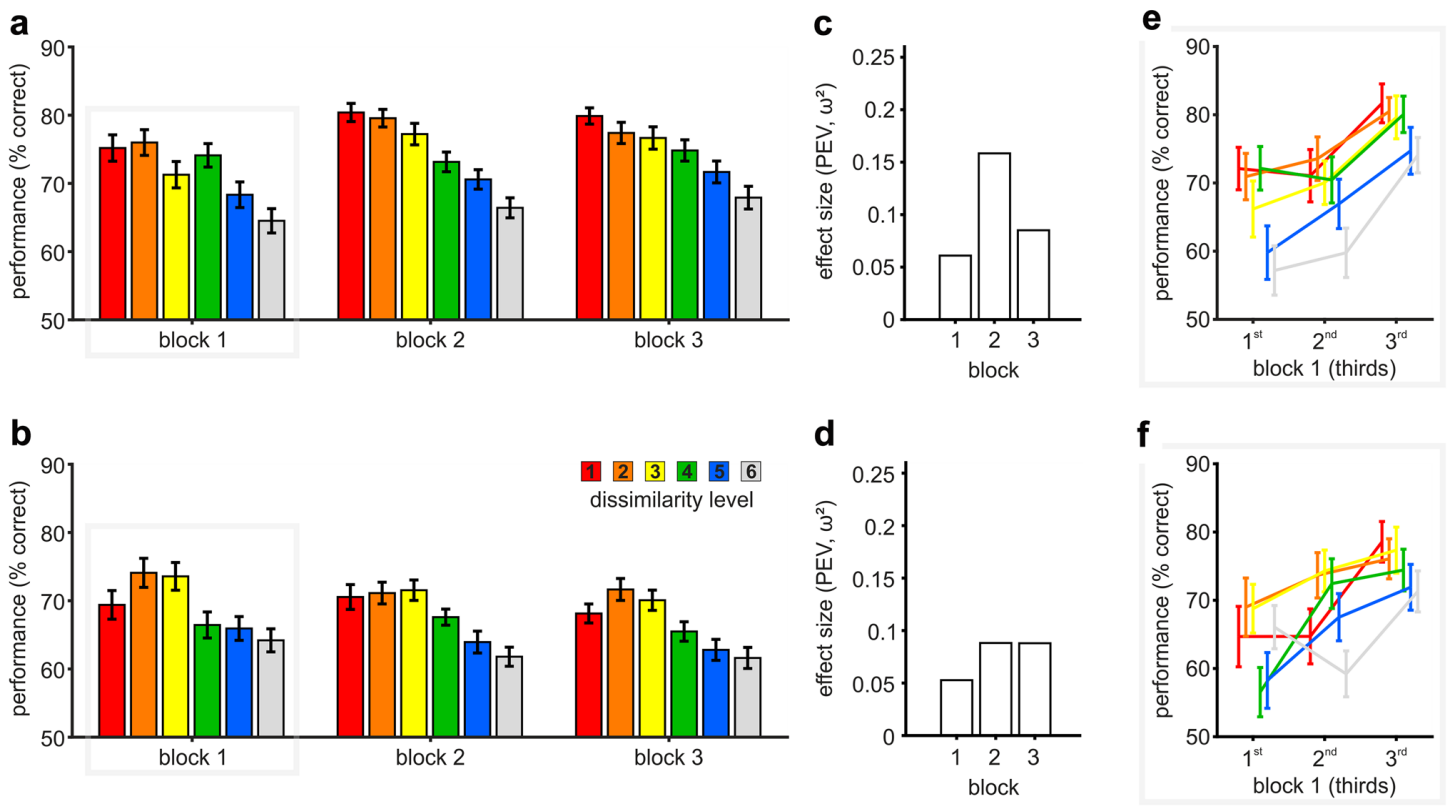
Performance in ‘exemplar’ sessions also depended on sample dissimilarity level. **a**, **b** Performance was dependent of sample dissimilarity level in each block within ‘exemplar’ sessions. Already the first block showed an overall decrease in performance due to increasing dissimilarity between sample and category prototype. **c**, **d** PEV values visualize the proportion of variance per block that can be explained by sample dissimilarity level. **e**, **f** Both birds showed an increase in average performance throughout the first block in ‘exemplar’ sessions for stimuli from all six levels. Unexpectedly, the performance of bird 1 exhibited level-dependent differences already within the first third of ‘exemplar’ sessions. The average performance was calculated per level within the first, second, and third part of block 1 in ‘exemplar’ sessions (mean ± SEM is shown). Bird 1 top row, *n* = 60 sessions; bird 2 bottom row, *n* = 52 sessions

Overall, we found that dissimilarity to the category prototype significantly affected categorization performance in ‘exemplar’ sessions (similar to ‘prototype’ sessions, Fig. 6 a & b right subplots, $F_{4,295} = 11.01,\, p < 0.0001, \,\omega^{2} = 0.1178,$ P(M|data) > 0.999, BF_M_ > 1000 and $F_{4,255} = 10.32, \,p < 0.0001, \,\omega^{2} = 0.1253,$, P(M|data) > 0.999, BF_M_ > 1000). The decline in performance in ‘prototype’ sessions had been larger, but nonetheless was also present and significant in ‘exemplar’ sessions (compare performance and effect size per dissimilarity level in ‘prototype’ and ‘exemplar’ sessions, Fig. 6a, b, e, f). The decrease of performance with increasing dissimilarity became more pronounced throughout ‘exemplar’ sessions (Fig. 8a, b), i.e., effect size increased over the first three blocks for bird 1 (0.0610, 0.1584, and 0.0851, for blocks 1, 2, and 3, respectively, Fig. 8c) and for bird 2 (0.0528, 0.0882, and 0.0880, for blocks 1, 2, and 3, respectively, Fig. 8d). In addition, sample dissimilarity level explained a higher percentage of performance variance compared to Δ-level (dissimilarity-level deviation between sample and match) already from the first block (Fig. 6e, f). For both birds and in each block, the relevance of dissimilarity level difference between sample and match was generally much smaller than that of sample and category prototype. Thus, already with limited experience with each category set, both birds successfully formed a characteristic internal representation of the underlying category prototype and used it for the decision of category membership. This suggests that both animals created a category prototype based on the different exemplars they encountered in ‘exemplar’ sessions to use the similarity relative to this one central stimulus for categorization.

However, it still remains unclear if the birds initially relied on exemplars and later switched to an internal prototype. To visualize the emergence of a dependency on an internal prototype, we analyzed the initial learning of each novel stimulus set focusing on the first block per ‘exemplar’ session. An increase in performance throughout the first block was apparent in the behavior of both birds (Fig. 8e, f). Both improved in categorizing novel RUBubble stimuli from all six dissimilarity levels. Interestingly, already within the first third of block 1, sample dissimilarity level differentially affected the performance of bird 1 ($F_{5,345} = 3.37, \,p = 0.0055, \,\omega^{2} = 0.0327, P\left( {\text{M|data}} \right) = 0.722, \,BF_{M} = 2.593$), but not for bird 2 ($F_{5,303} = 1.86, \,p = 0.1005, \,\omega^{2} = 0.0138, P\left( {\text{M|data}} \right) = 0.138, \,BF_{M} = 0.160$).

## Discussion

We trained two jackdaws on a delayed match to category paradigm using a novel, artificial stimulus type, RUBubbles. Both birds learned to differentiate between two session-unique categories following a prototype- or exemplar-based learning approach. Performance in either session type depended most on the similarity between sample and category prototype, suggesting a prototype-based strategy for categorization. Overall, our birds appeared to rely on a central category prototype, irrespective of the learning approach. More precisely, the similarity to the prototype explained categorization performance best, regardless of if a prototype was directly presented (as the choice stimulus in ‘prototype’ sessions) or had to be inferred based on a subset of exemplar stimuli (‘exemplar’ sessions). This preference for a specific strategy could potentially be ascribed to our stimulus set composition or could reflect a general bias towards prototype category representations.

### Stimulus variability affected initial learning but not generalization performance

Prototype- and exemplar-based sessions differed regarding the stimulus composition; however, behavioral results from the two learning procedures were surprisingly similar. The full range of category members was presented already within the first trials of ‘exemplar’ sessions (i.e., samples from all dissimilarity levels). Such higher stimulus variability was reported to “help in identifying task-relevant dimensions and establishing correct decision boundaries” (Raviv et al. 2022, p. 473), which can facilitate categorization (Hahn et al. 2005; Wahlheim et al. 2012). Therefore, we expected a higher generalization performance (i.e., a better performance for more diverging sample stimuli) and a generally more flexible, overarching category representation arising from exemplar-based learning. Highly variable (and thus potentially more representative) input was shown to result in a more general and robust generalization performance facilitating more abstract representations (Posner and Keele 1968), however, at the cost of slower initial learning (Raviv et al. 2022). For example, a beneficial effect of stimulus set variability was previously reported in children (Xu and Tenenbaum 2007; Mather and Plunkett 2011). In contrast, less variable input allows fast initial learning but reduced generalization due to narrow category inclusion boundaries (Raviv et al. 2022). In our prototype approach, the birds experienced category boundaries successively moving outwards from a central prototype (i.e., via the increase in sample dissimilarity level relative to the prototype, Fig. 3). Thus, they constantly had to update their presumed category boundaries (or the lowest level of similarity that still represented the same category) to incorporate more diverging category members. We found faster initial learning due to lower variability in both birds (‘prototype’ sessions). However, only one bird showed a minor beneficial effect on subsequent generalization performance (following high stimulus variability in ‘exemplar’ sessions). The complete lack of this observation in the other bird could potentially be attributed to its overall higher performance on either session type (Fig. 6 top).

### Prototype-based categorization results from previous categorization experience and category structure

Another factor influencing categorization behavior is previous experience. Our birds were trained for a prolonged period to categorize RUBubble stimuli (with novel sets per session) and thus most likely learned to ignore stimulus identity in favor of stimulus category (Bowman et al. 2020). Ignoring stimulus identity argues against an exemplar-based category representation which would require an identification of individual category members (Nosofsky 1987). Initial training on RUBubble categories involved only prototype-based sessions with the exemplar-based protocol as secondary task variation. This could have biased our birds towards a prototype-based categorization strategy. Furthermore, category structure has a substantial influence, showing a clear advantage of prototype representations for perceptually coherent categories (Smith et al. 2008; Smith 2014; Bowman and Zeithamova 2020). Our RUBubble categories were created from one central category prototype per category, using increasingly diverging parameter ranges (see Fig. 1). Thus, they were always (by design) defined in terms of graded perceptual similarity and members could be classified purely based on similarity to the respective category prototype. This inherent category structure was similar in ‘prototype’ and ‘exemplar’ sessions. The close perceptual similarity within some RUBubble category sets could further have discouraged our birds to differentiate individual category members (Smith et al. 2008).

The number of category members can also influence which strategy is favored. A large number of complex stimuli support a prototype-based strategy (Jitsumori and Delius 2001; Minda and Smith 2001). We overall used the same number of unique RUBubble stimuli in both ‘prototype’ and ‘exemplar’ sessions (distinct blocks with slightly differing stimulus numbers, see Fig. 3), which quickly made memorization of individual stimuli very demanding. Therefore, the stimulus sets we used, together with the birds’ training history, could have biased our birds to adopt a prototype-based strategy. This might also explain why we found no effect of stimulus familiarity in general (not even at the beginning of ‘exemplar’ sessions, when the low number of observed stimuli would have made such a strategy feasible; Fig. S6). Consequently, it was both possible and the most effective strategy to construct a category prototype in ‘exemplar’ sessions (something the birds potentially learned during their prolonged training experience).

We did not implement a model-based approach to analyze our results. Due to the high number of dimensions in our stimulus design (i.e., with respect to (dis-) similarity of stimuli to their respective category prototype, and to the prototype of the alternative category), such an approach turned out as not feasible. With appropriate changes in stimulus design, a model-based analysis could give us more detailed information, in particular about exemplar-based categorization. This line of analysis will be required in the future to comprehensively address questions about differences between potential prototype- and exemplar-based strategies.

### Prototype representations as default strategy?

Categorization of information that has to be encoded and maintained in memory helps to efficiently reduce the processing amount and effectively mitigates the effect of noise on working memory representations (Olsson and Poom 2005; Panichello et al. 2019). If the details of individual items are irrelevant to guide future behavior, it would be advantageous to focus only on the diagnostic aspects of stimuli that identify their category (Olsson and Poom 2005; Smith et al. 2010; Smith 2014). Our results suggest that when confronted with few trials of only a subset of category exemplars, both birds formed an approximation of the category prototype, focusing on the overall category instead of individual stimuli. Thus, they quickly adopted the most efficient strategy to categorize our artificial RUBubble categories. The prototype advantage we found could indicate a general bias towards prototype representations that goes beyond the detailed aspects of our stimulus set. Most ecologically relevant categories follow a similarity-based or family-resemblance structure (e.g., (Smith et al. 2010)). Therefore, defaulting to prototype-based representations is likely adaptive over rote learning of myriads of individual exemplars. An almost instantaneously emerging prototype representation based on the experience of only few category exemplars as we have found with our jackdaws has been shown before (Smith 2014; Tiedemann et al. 2022). Prototype representation as a default and most efficient categorization strategy has been reported in primates (Smith et al. 2008, 2010), pigeons (Cook and Smith 2006), humans (Minda and Smith 2001; Smith and Minda 2002; Cook and Smith 2006; Smith et al. 2010; Bowman et al. 2020), and even formal computer simulations (Smith 2014). This led to the conclusion that a default prototype representation might be present in several vertebrate evolutionary lines (Smith et al. 2016).

A potential issue concerning categorization abilities shown for various animal species is that they have often relied on training of specific categories across multiple sessions. For example, following extensive training, excellent categorization abilities in pigeons were shown to range from similarity-based categorization to the formation of abstract concepts (e.g., (Levenson et al. 2015; Peissig et al. 2019); Picasso vs. Monet paintings: (Watanabe et al. 1995; Anderson et al. 2020), numerosity: (Scarf et al. 2011), same–different: (Katz and Wright 2006), word non-word orthographic processing: (Scarf et al. 2016), behavioral meaning: (Kirsch et al. 2009), concept ‘human’: (Herrnstein and Loveland 1964)). Our experimental approach required learning to categorize complex artificial stimuli within individual sessions. Therefore, we decided to probe categorization learning in jackdaws (members of the corvid family, excellent at mastering cognitively challenging categorization tasks: (Veit and Nieder 2013; Ditz and Nieder 2016)). We can now add successful categorization of arbitrary and complex stimuli in single experimental sessions to the cognitive capabilities of corvids. In our study, a prototype-based categorization occurred already after very short exposure to novel categories, mirroring findings in humans and computer simulations (e.g., (Smith 2014; Tiedemann et al. 2022)). Our behavioral analyses were limited by our stimulus design that required us to drastically reduce complexity. Thus, we might not have been able to sufficiently differentiate between individual stimuli to detect an exemplar representation of the very first few stimuli. We therefore advise caution to rule out exemplar-based processes. Investigating categorization in jackdaws offers the advantage of providing a valuable comparative perspective enabling us to study categorization strategies in highly visual animals whose brain evolution differs profoundly from mammals (for example with regard to the organization of pallial regions into nuclei instead of the layered neocortex (Clark and Colombo 2020)). Examining the neuronal basis supporting categorization learning would be highly informative, for instance to resolve the interplay of prototype- and exemplar-based processes, which has been studied in humans (Bowman et al. 2020). The visual system of birds has gathered substantial interest in this regard as it offers a clear structure and hierarchy of processing stimuli for categorization (Pusch et al. 2022). In humans, different brain areas within the categorization network were shown to be involved in either prototype (e.g., ventromedial prefrontal cortex and anterior hippocampus) or exemplar representations (e.g., inferior frontal gyrus and lateral parietal cortex) (Bowman et al. 2020). Bowman et al. (2020) were able to identify both representations in a single study, however, they found a clear prototype advantage in the final task. Yet, some authors have also argued for exemplar representation as a secondary process, that would be shaped following sufficient previous experience with the category (e.g., (Minda and Smith 2001)) and thus only found later on. Nevertheless, additional electrophysical recordings within the avian categorization network could further our understanding of categorization strategies in jackdaws to investigate the existence of (prospective) encoding of category prototypes or individual representations of specific exemplars.

### Category exceptions and stimulus identification may encourage non-prototype strategy

Two potential changes in our experimental design could be implemented to mitigate the bias towards a prototype-based strategy in future experiments. Prototype representations are based on similarity relations, characterized by indistinct category boundaries, and thus largely unsuitable for correct exception classification (Cook and Smith 2006; Smith et al. 2010, 2016). Introducing exception stimuli whose category membership must be learned by rote could be used to weaken the bias towards a prototype representation in our paradigm by favoring an exemplar-based approach (although previous studies in monkeys failed to bias their subjects reliably towards exemplar-based strategies with only a few stimuli (prototype-exception task) (Smith et al. 2010)). Exemplar memorization could further be enhanced by introducing additional probe trials focusing on the working memory representation of distinct stimuli (i.e., delayed match to sample trials with both choice stimuli matching the category), reducing the number of stimuli, or repeatedly present individual sample stimuli (Smith et al. 2008).

With our stimulus generation, we have controlled the similarity relative to the corresponding category prototype (via the differentiation of specific dissimilarity levels); however, the similarity of individual stimuli to the other category prototype remains ambiguous (see Fig. 1d). This complicates the interpretation of results and the application of categorization models. One possible solution for future studies could be the use of a continuous category created from two RUBubble stimuli serving as category prototypes. In this continuum, a decreasing similarity to category A would always be linked to an increasing similarity relative to category B (Apostel and Rose 2021). By removing stimuli equidistant to both category prototypes, one could then create a clear boundary despite the initial continuum.

## Conclusion

We showed that learning of novel, artificial categories was dependent on the specific experience with individual category stimuli. Jackdaws consistently used a central prototype to judge category membership, regardless of whether this prototype was used to introduce distinct categories or had to be created from multiple exemplars. This default prototype assumption in early categorization learning was similar to earlier findings in various human and animal subjects (monkeys, pigeons), and might reflect a predominant category structure. Just as humans and pigeons, jackdaws might exhibit an additional exemplar representation of individual stimuli if required by the specific situation. Generally, the behavior of jackdaws reflected a central category prototype as the most adaptive, efficient, and parsimonious way to represent RUBubble categories (in agreement with the specific task demands).

### Supplementary Information

Below is the link to the electronic supplementary material.

Supplementary file1 (DOCX 885 KB)

## Data Availability

The datasets generated and analyzed during the current study are available from the corresponding author on reasonable request.
