# Supplementary material for: Jackdaws form categorical prototypes based on experience with category exemplars
Source: Brain Struct Funct. 2023 Jun 1;229(3):593–608. doi: 10.1007/s00429-023-02651-w (PMC10978630; doi:10.1007/s00429-023-02651-w)
Supplement: Supplementary file 1 — Supplementary file1 (DOCX 885 KB) [file 429_2023_2651_MOESM1_ESM.docx]

**Jackdaws form categorical prototypes based on experience with category exemplars**

**Brain Structure and Function**

**Aylin Apostel^1^, Lukas Alexander Hahn^1^ & Jonas Rose^1^**

*^1^Neural Basis of Learning, Institute of Cognitive Neuroscience, Faculty of Psychology, Ruhr University Bochum, 44801 Bochum, Germany*

*Correspondence:* [*jonas.rose@ruhr-uni-bochum.de*](mailto:jonas.rose@ruhr-uni-bochum.de) *(J.R.)*


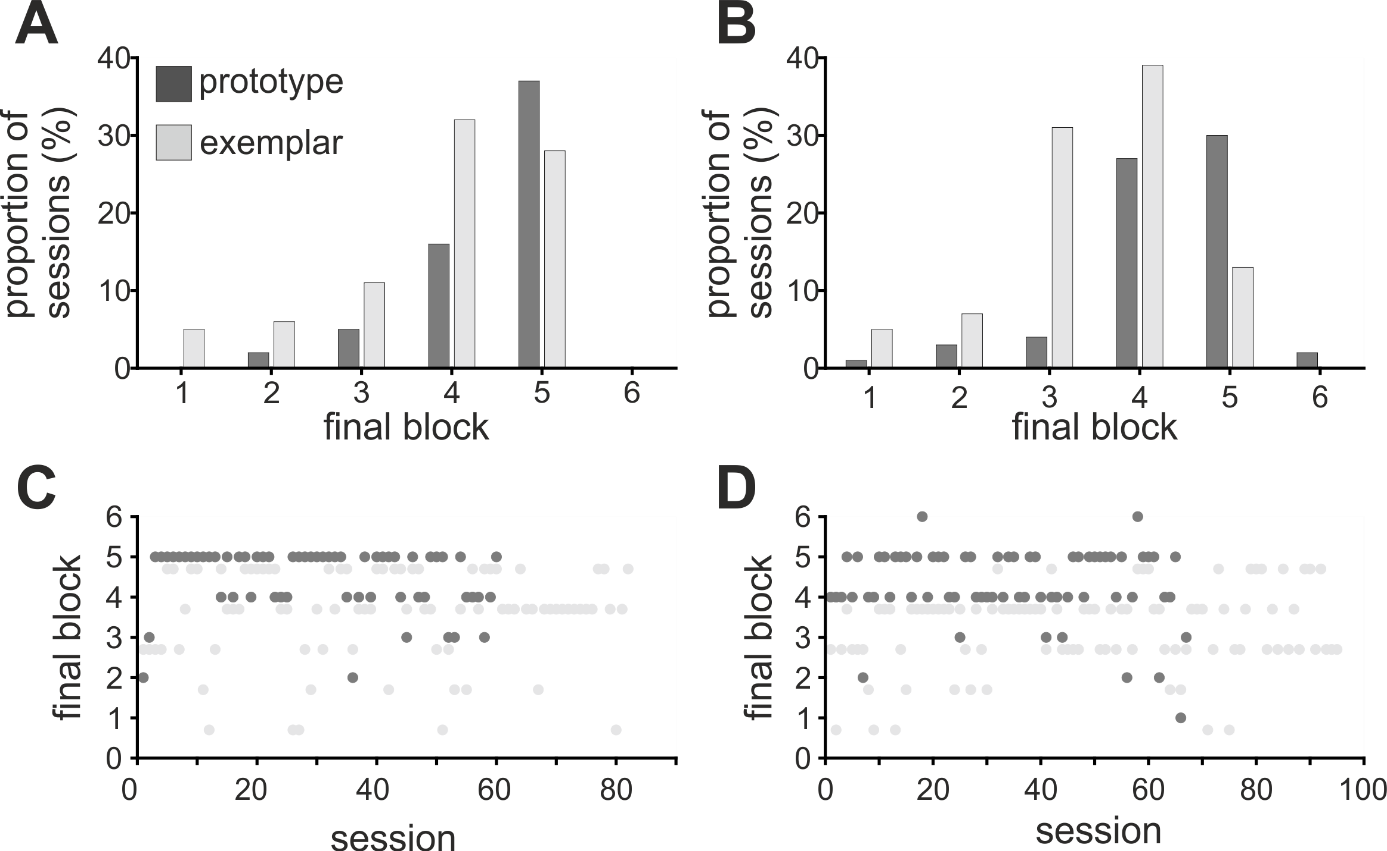


**Figure S1:** Overview of all experimental sessions. **A, B** Percentage of sessions in which the birds reached a specific number of blocks. **C, D** Chronological overview of all sessions visualizing which block was reached in which session. Generally, both birds reached the higher blocks in most experimental sessions. **A, C:** bird 1, **B, D:** bird 2.


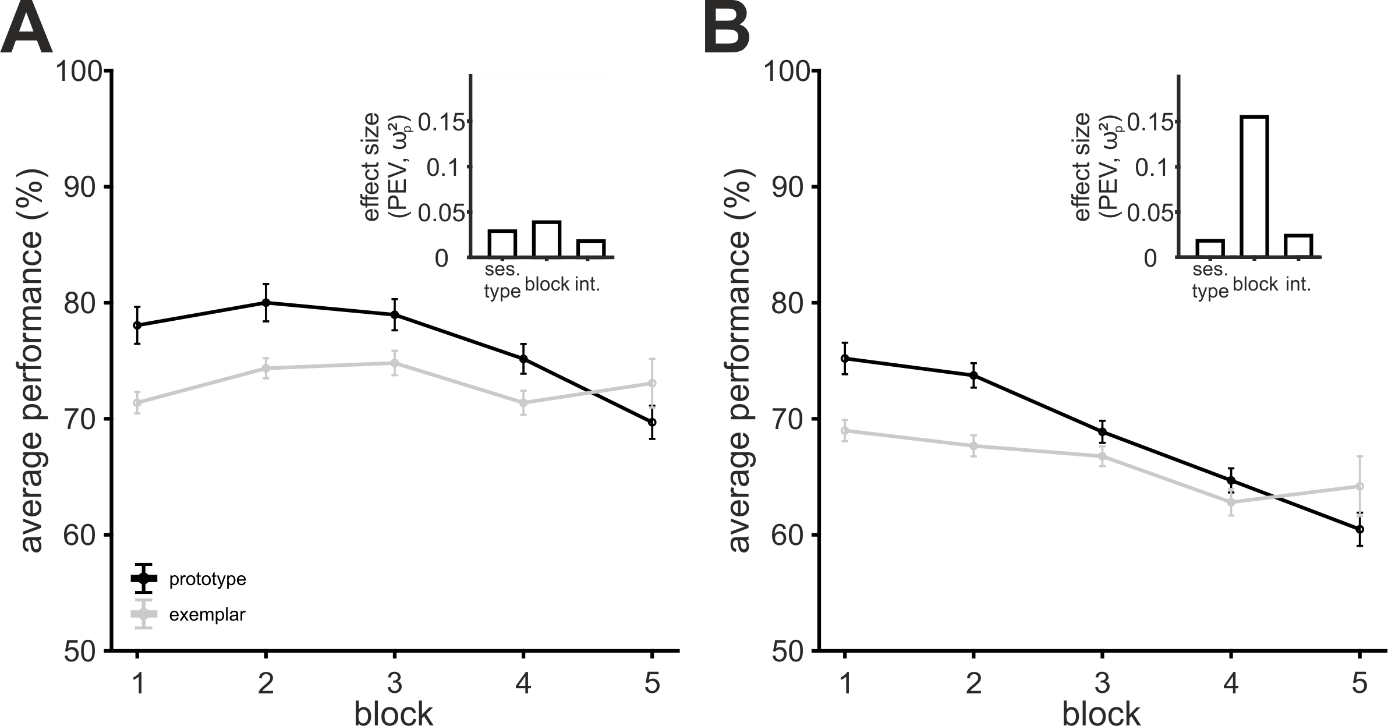


**Figure S2:** Performance decreased towards the end of a session (indicated by higher block numbers). This decrement was more pronounced for ‘prototype’ sessions. We tested the influence of block (a proxy for the temporal component of a session), with a two-way ANOVA with factors session type (‘prototype’ or ‘exemplar’), block (1-5), and their interaction. We found for both birds a significant decline of performance with block (bird 1: F(4,507) = 6.21, p < 0.0001, $\omega_{p}^{2}$ = 0.0387; bird 2: F(4,479) = 23.47, p < 0.0001, $\omega_{p}^{2}$ = 0.1553), and a significant effect of training approach (bird 1: F(1,507) = 16.37, p < 0.0001, $\omega_{p}^{2}$ = 0.0289; bird 2: F(1,479) = 10.03, p = 0.0016, $\omega_{p}^{2}$ = 0.0181). There was also a significant interaction (bird 1: F(4,507) = 3.36, p = 0.0099, $\omega_{p}^{2}$ = 0.0179; bird 2: F(4,479) = 3.99, p = 0.0034, $\omega_{p}^{2}$ = 0.0239). A Bayesian ANOVA (with the same factors, each model factor at prior P(M) = 0.2) revealed that for bird 1, a model incorporating both block and session type, and their interaction was most likely to explain the data (P(B+T+BxT|data) = 0.593, BF_M_ = 5.828), followed by a model with both factors but without the interaction (P(B+T|data) = 0.406, BF_M_ = 2.737), and models with only one factor or the null model (all P(M|data)<0.001, all BF_M_ ≤ 0.003). The same was true for bird 2 (interaction model P(B+T+BxT|data) = 0.952, BF_M_ = 78.817; factors without interaction P(B+T|data) = 0.048, BF_M_ = 0.203; all other P(M|data) < 0.001, BF_M_ < 0.001). Thus, effects tied to the block structure (like diminishing motivation due to saturation) were present for both types of sessions. Insets show corresponding effect sizes of the two-way ANOVA. Overall, the results indicate a strong influence of block number on performance that is dependent on the session type. Due to the significant interaction, we further analyzed the individual effects per session type for each bird performing a one-way ANOVA. **A** Bird 1 showed an effect of block number on performance that reached significance for ‘prototype’ (F(4,244) = 6.67, p < 0.0001, ω² = 0.0835, P(block|data) = 0.997, BF_M_ = 354.973) and ‘exemplar’ sessions (F(4,263) = 2.42, p = 0.0486, ω² = 0.0208, P(block|data) = 0.649, BF_M_ = 1.850). However, post-hoc comparisons of blocks revealed that for ‘prototype’ sessions the significant difference arose from performance in block 5 differing from blocks 1 to 3 (all p < 0.05, Tab. S1), without significant differences between adjacent blocks (all p > 0.05). In ‘exemplar’ sessions, no direct comparisons of block performances became significant (all p > 0.05). **B** Bird 2 showed a significant decrease of performance for both training approaches (‘prototype’ sessions: F(4,263) = 24.22, p < 0.0001, ω² = 0.2574, P(block|data) > 0.999, BF_M_ > 1000; ‘exemplar’ sessions: F(4,216) = 5.81, p = 0.0002, ω² = 0.0801, P(block|data) = 0.992, BF_M_ = 127.960), with ‘prototype’ sessions being more affected by block number. Post-hoc comparisons further indicated that performance significantly decreased between adjacent blocks of ‘prototype’ sessions and for some comparisons in ‘exemplar’ sessions (Tab. S1).

**Table S1:** Overview of post-hoc comparisons of the results from the one-way ANOVA with factor ‘block’, calculated per session type (i.e., ‘prototype’, ‘exemplar’) and bird using corrected alpha-values.

| **Bird 1** | **Block comparison** | | **p-value** | **Bird 2** | **Block comparison** | | **p-value** |
| --- | --- | --- | --- | --- | --- | --- | --- |
| prototype  sessions | 1 | 2 | 0.8704 | prototype  sessions | 1 | 2 | 0.8832 |
|  | 1 | 3 | 0.9914 |  | 1 | 3 | 0.0005 |
|  | 1 | 4 | 0.6074 |  | 1 | 4 | < 0.0001 |
|  | 1 | 5 | 0.0016 |  | 1 | 5 | < 0.0001 |
|  | 2 | 3 | 0.9860 |  | 2 | 3 | 0.0159 |
|  | 2 | 4 | 0.1160 |  | 2 | 4 | < 0.0001 |
|  | 2 | 5 | < 0.0001 |  | 2 | 5 | < 0.0001 |
|  | 3 | 4 | 0.3263 |  | 3 | 4 | 0.0568 |
|  | 3 | 5 | 0.0003 |  | 3 | 5 | < 0.0001 |
|  | 4 | 5 | 0.1013 |  | 4 | 5 | 0.1564 |
| exemplar  sessions | 1 | 2 | 0.2511 | exemplar  sessions | 1 | 2 | 0.8783 |
|  | 1 | 3 | 0.1311 |  | 1 | 3 | 0.5036 |
|  | 1 | 4 | 0.9999 |  | 1 | 4 | < 0.0001 |
|  | 1 | 5 | 0.8899 |  | 1 | 5 | 0.1876 |
|  | 2 | 3 | 0.9979 |  | 2 | 3 | 0.9680 |
|  | 2 | 4 | 0.2486 |  | 2 | 4 | 0.0044 |
|  | 2 | 5 | 0.9562 |  | 2 | 5 | 0.5104 |
|  | 3 | 4 | 0.1295 |  | 3 | 4 | 0.0359 |
|  | 3 | 5 | 0.8765 |  | 3 | 5 | 0.7668 |
|  | 4 | 5 | 0.8883 |  | 4 | 5 | 0.9709 |

**Tab. S2:** Example of the approach to detect differences in the learning rates (slopes of Fig. 5b). Total counts of correct and false categorization (cumulative of trials 1 to 5), across all sessions of animal 1. Observed values, [expected values if non-different], $\chi_{(1)}^{2}=16.58, p<0.0001$.

| Bin 1 (trial 1 to 5) | **Prototype** | **Exemplar** | **Σ** |
| --- | --- | --- | --- |
| **Correct** | 210 [188.1] | 191 [212.9] | 401 |
| **Error** | 55 [76.92] | 109 [87.1] | 164 |
| **Σ** | 265 | 300 | 565 |

**Table S3**: Statistical results of the $\chi^{2}$ test analyzing differences in the learning curve slopes between ‘prototype’ and ‘exemplar’ sessions. We found significant differences only at the beginning of the first 100 trials (criteria for significance were two-consecutive, non-overlapping bins, individually significant at an alpha of 0.05).

|  | **first 100** | | | **last 100** | | |  | **first 100** | | | **last 100** | | |
| --- | --- | --- | --- | --- | --- | --- | --- | --- | --- | --- | --- | --- | --- |
|  | $\boldsymbol{\chi}^{\boldsymbol{2}}$ | **df** | **p** | $\boldsymbol{\chi}^{\boldsymbol{2}}$ | **df** | **p** |  | $\boldsymbol{\chi}^{\boldsymbol{2}}$ | **df** | **p** | $\boldsymbol{\chi}^{\boldsymbol{2}}$ | **df** | **p** |
| **bird 1** | 16.576 | 1 | <0.0001 | 1.5562 | 1 | 0.2122 | **bird 2** | 20.898 | 1 | 0 | 0.9979 | 1 | 0.3178 |
|  | 14.262 | 1 | 0.0002 | 1.6462 | 1 | 0.1995 |  | 15.516 | 1 | 0.0001 | 0.0479 | 1 | 0.8268 |
|  | 9.1519 | 1 | 0.0025 | 1.3855 | 1 | 0.2392 |  | 8.0961 | 1 | 0.0044 | 1.194 | 1 | 0.2745 |
|  | 5.6633 | 1 | 0.0173 | 0.0025 | 1 | 0.9597 |  | 3.1139 | 1 | 0.0776 | 0.261 | 1 | 0.6094 |
|  | 1.7895 | 1 | 0.181 | 0.0064 | 1 | 0.9364 |  | 5.3279 | 1 | 0.021 | 1.9669 | 1 | 0.1608 |
|  | 3.2645 | 1 | 0.0708 | 0.0366 | 1 | 0.8483 |  | 9.1664 | 1 | 0.0025 | 0.1464 | 1 | 0.702 |
|  | 1.9689 | 1 | 0.1606 | 0.0005 | 1 | 0.9824 |  | 19.318 | 1 | 0 | 0 | 1 | 0.9949 |
|  | 3.5764 | 1 | 0.0586 | 0.1054 | 1 | 0.7454 |  | 16.241 | 1 | 0.0001 | 0.0004 | 1 | 0.9833 |
|  | 6.6704 | 1 | 0.0098 | 0.3949 | 1 | 0.5297 |  | 12.129 | 1 | 0.0005 | 0.0004 | 1 | 0.9833 |
|  | 5.9981 | 1 | 0.0143 | 0.2744 | 1 | 0.6004 |  | 6.9803 | 1 | 0.0082 | 0.2267 | 1 | 0.634 |
|  | 5.9981 | 1 | 0.0143 | 0.4885 | 1 | 0.4846 |  | 2.2455 | 1 | 0.134 | 0.1411 | 1 | 0.7072 |
|  | 7.509 | 1 | 0.0061 | 0.374 | 1 | 0.5408 |  | 0.1868 | 1 | 0.6656 | 0.4511 | 1 | 0.5018 |
|  | 10.324 | 1 | 0.0013 | 0.3917 | 1 | 0.5314 |  | 0.6217 | 1 | 0.4304 | 0.1227 | 1 | 0.7262 |
|  | 5.7589 | 1 | 0.0164 | 1.1128 | 1 | 0.2915 |  | 4.1608 | 1 | 0.0414 | 0.0368 | 1 | 0.8478 |
|  | 7.6798 | 1 | 0.0056 | 0.686 | 1 | 0.4075 |  | 3.0856 | 1 | 0.079 | 0.07 | 1 | 0.7914 |
|  | 6.4766 | 1 | 0.0109 | 0.202 | 1 | 0.6532 |  | 3.4576 | 1 | 0.063 | 0.0693 | 1 | 0.7923 |
|  | 5.17 | 1 | 0.023 | 0.2359 | 1 | 0.6272 |  | 2.1921 | 1 | 0.1387 | 0.1841 | 1 | 0.6679 |
|  | 4.6982 | 1 | 0.0302 | 1.0186 | 1 | 0.3128 |  | 2.4267 | 1 | 0.1193 | 0.3231 | 1 | 0.5698 |
|  | 9.7097 | 1 | 0.0018 | 5.0188 | 1 | 0.0251 |  | 0.9492 | 1 | 0.3299 | 0.5342 | 1 | 0.4649 |
|  | 10.225 | 1 | 0.0014 | 1.8529 | 1 | 0.1734 |  | 1.1246 | 1 | 0.2889 | 0.506 | 1 | 0.4769 |
|  | 12.684 | 1 | 0.0004 | 2.1984 | 1 | 0.1382 |  | 3.8174 | 1 | 0.0507 | 2.0061 | 1 | 0.1567 |
|  | 14.473 | 1 | 0.0001 | 1.292 | 1 | 0.2557 |  | 3.1012 | 1 | 0.0782 | 1.3117 | 1 | 0.2521 |
|  | 6.1889 | 1 | 0.0129 | 0.8598 | 1 | 0.3538 |  | 0.8058 | 1 | 0.3694 | 4.0271 | 1 | 0.0448 |
|  | 2.8638 | 1 | 0.0906 | 0.1116 | 1 | 0.7383 |  | 1.4494 | 1 | 0.2286 | 8.5441 | 1 | 0.0035 |
|  | 1.7373 | 1 | 0.1875 | 0.0658 | 1 | 0.7976 |  | 3.2224 | 1 | 0.0726 | 6.7688 | 1 | 0.0093 |
|  | 0.7616 | 1 | 0.3828 | 3.0928 | 1 | 0.0786 |  | 1.7322 | 1 | 0.1881 | 3.6485 | 1 | 0.0561 |
|  | 0.1031 | 1 | 0.7482 | 7.3351 | 1 | 0.0068 |  | 4.2855 | 1 | 0.0384 | 2.7265 | 1 | 0.0987 |
|  | 2.2756 | 1 | 0.1314 | 8.7437 | 1 | 0.0031 |  | 10.886 | 1 | 0.001 | 0.0181 | 1 | 0.8931 |
|  | 5.5861 | 1 | 0.0181 | 5.8568 | 1 | 0.0155 |  | 8.5095 | 1 | 0.0035 | 1.5913 | 1 | 0.2071 |
|  | 2.0374 | 1 | 0.1535 | 5.4615 | 1 | 0.0194 |  | 5.9226 | 1 | 0.0149 | 2.9558 | 1 | 0.0856 |
|  | 2.1826 | 1 | 0.1396 | 1.1255 | 1 | 0.2887 |  | 6.9104 | 1 | 0.0086 | 3.2081 | 1 | 0.0733 |
|  | 3.0028 | 1 | 0.0831 | 1.0783 | 1 | 0.2991 |  | 9.2196 | 1 | 0.0024 | 7.1535 | 1 | 0.0075 |
|  | 0.1055 | 1 | 0.7453 | 0.2892 | 1 | 0.5907 |  | 5.4303 | 1 | 0.0198 | 2.5981 | 1 | 0.107 |
|  | 0.0611 | 1 | 0.8048 | 0.0552 | 1 | 0.8142 |  | 7.4393 | 1 | 0.0064 | 1.8574 | 1 | 0.1729 |
|  | 1.7416 | 1 | 0.1869 | 0.0658 | 1 | 0.7976 |  | 3.7421 | 1 | 0.0531 | 1.3061 | 1 | 0.2531 |
|  | 2.2716 | 1 | 0.1318 | 0.3776 | 1 | 0.5389 |  | 3.4001 | 1 | 0.0652 | 0.2324 | 1 | 0.6298 |
|  | 4.0847 | 1 | 0.0433 | 0.9878 | 1 | 0.3203 |  | 2.4267 | 1 | 0.1193 | 0.0525 | 1 | 0.8189 |
|  | 7.8334 | 1 | 0.0051 | 0.2455 | 1 | 0.6203 |  | 7.4484 | 1 | 0.0063 | 1.0131 | 1 | 0.3142 |
|  | 5.6318 | 1 | 0.0176 | 0.14 | 1 | 0.7083 |  | 5.9885 | 1 | 0.0144 | 2.3285 | 1 | 0.127 |
|  | 5.6318 | 1 | 0.0176 | 0.0409 | 1 | 0.8396 |  | 14.17 | 1 | 0.0002 | 1.2099 | 1 | 0.2714 |
|  | 6.8918 | 1 | 0.0087 | 0.0331 | 1 | 0.8556 |  | 12.766 | 1 | 0.0004 | 2.1426 | 1 | 0.1433 |
|  | 1.6562 | 1 | 0.1981 | 0.7445 | 1 | 0.3882 |  | 4.4359 | 1 | 0.0352 | 2.6216 | 1 | 0.1054 |
|  | 1.0657 | 1 | 0.3019 | 0.0005 | 1 | 0.9824 |  | 2.0745 | 1 | 0.1498 | 0.167 | 1 | 0.6828 |
|  | 2.9929 | 1 | 0.0836 | 0.1804 | 1 | 0.671 |  | 0.577 | 1 | 0.4475 | 0.2735 | 1 | 0.601 |
|  | 3.3945 | 1 | 0.0654 | 0.0152 | 1 | 0.9018 |  | 0.0278 | 1 | 0.8675 | 0.0255 | 1 | 0.8731 |
|  | 2.4003 | 1 | 0.1213 | 0.0011 | 1 | 0.9731 |  | 0.0164 | 1 | 0.898 | 0.429 | 1 | 0.5125 |
|  | 6.5782 | 1 | 0.0103 | 1.1128 | 1 | 0.2915 |  | 3.506 | 1 | 0.0611 | 1.2072 | 1 | 0.2719 |
|  | 7.3377 | 1 | 0.0068 | 0.189 | 1 | 0.6638 |  | 4.6617 | 1 | 0.0308 | 2.5525 | 1 | 0.1101 |
|  | 5.1199 | 1 | 0.0237 | 0.1054 | 1 | 0.7454 |  | 4.2855 | 1 | 0.0384 | 2.7634 | 1 | 0.0964 |
|  | 5.245 | 1 | 0.022 | 0.5221 | 1 | 0.4699 |  | 4.2692 | 1 | 0.0388 | 1.8101 | 1 | 0.1785 |
|  | 3.4745 | 1 | 0.0623 | 0.4604 | 1 | 0.4974 |  | 1.1608 | 1 | 0.2813 | 0.0188 | 1 | 0.891 |
|  | 0.6886 | 1 | 0.4067 | 1.1996 | 1 | 0.2734 |  | 0.0072 | 1 | 0.9322 | 1.0235 | 1 | 0.3117 |
|  | 0.7875 | 1 | 0.3748 | 0.3947 | 1 | 0.5298 |  | 1.0191 | 1 | 0.3127 | 0.5898 | 1 | 0.4425 |
|  | 0.5524 | 1 | 0.4573 | 0.8355 | 1 | 0.3607 |  | 0.4507 | 1 | 0.502 | 0.7135 | 1 | 0.3983 |
|  | 0.6909 | 1 | 0.4059 | 0.1526 | 1 | 0.6961 |  | 0.305 | 1 | 0.5808 | 0.9649 | 1 | 0.326 |
|  | 1.1055 | 1 | 0.2931 | 0.2264 | 1 | 0.6342 |  | 0.2631 | 1 | 0.608 | 0.0765 | 1 | 0.7821 |
|  | 4.4291 | 1 | 0.0353 | 0.2037 | 1 | 0.6518 |  | 0.3886 | 1 | 0.533 | 0.3432 | 1 | 0.558 |
|  | 7.2107 | 1 | 0.0072 | 0.8577 | 1 | 0.3544 |  | 1.9619 | 1 | 0.1613 | 0.1426 | 1 | 0.7057 |
|  | 10.788 | 1 | 0.001 | 1.4211 | 1 | 0.2332 |  | 2.017 | 1 | 0.1555 | 1.5494 | 1 | 0.2132 |
|  | 9.868 | 1 | 0.0017 | 1.6269 | 1 | 0.2021 |  | 1.7504 | 1 | 0.1858 | 1.066 | 1 | 0.3019 |
|  | 10.777 | 1 | 0.001 | 0.4527 | 1 | 0.5011 |  | 0.3051 | 1 | 0.5807 | 3.8492 | 1 | 0.0498 |
|  | 8.3487 | 1 | 0.0039 | 0.0892 | 1 | 0.7652 |  | 1.2377 | 1 | 0.2659 | 2.744 | 1 | 0.0976 |
|  | 10.41 | 1 | 0.0013 | 0.498 | 1 | 0.4804 |  | 1.2545 | 1 | 0.2627 | 1.3489 | 1 | 0.2455 |
|  | 7.3562 | 1 | 0.0067 | 2.7067 | 1 | 0.0999 |  | 0.4005 | 1 | 0.5268 | 0.7045 | 1 | 0.4013 |
|  | 6.7524 | 1 | 0.0094 | 3.3774 | 1 | 0.0661 |  | 0.0431 | 1 | 0.8356 | 1.4997 | 1 | 0.2207 |
|  | 8.1573 | 1 | 0.0043 | 5.2408 | 1 | 0.0221 |  | 0.6355 | 1 | 0.4254 | 0.0129 | 1 | 0.9094 |
|  | 9.868 | 1 | 0.0017 | 3.8947 | 1 | 0.0484 |  | 0.1098 | 1 | 0.7404 | 1.1507 | 1 | 0.2834 |
|  | 3.5608 | 1 | 0.0592 | 3.5421 | 1 | 0.0598 |  | 0.2838 | 1 | 0.5942 | 0.0092 | 1 | 0.9234 |
|  | 2.51 | 1 | 0.1131 | 0.9316 | 1 | 0.3345 |  | 1.2377 | 1 | 0.2659 | 0.1642 | 1 | 0.6853 |
|  | 4.4291 | 1 | 0.0353 | 0.208 | 1 | 0.6483 |  | 0.1556 | 1 | 0.6933 | 0.0385 | 1 | 0.8445 |
|  | 4.1572 | 1 | 0.0415 | 0.7089 | 1 | 0.3998 |  | 0.0526 | 1 | 0.8186 | 0.2371 | 1 | 0.6263 |
|  | 6.9325 | 1 | 0.0085 | 2.0676 | 1 | 0.1505 |  | 1.3061 | 1 | 0.2531 | 0.0301 | 1 | 0.8622 |
|  | 7.1843 | 1 | 0.0074 | 0.1463 | 1 | 0.7021 |  | 0.327 | 1 | 0.5674 | 1.3173 | 1 | 0.2511 |
|  | 6.2697 | 1 | 0.0123 | 0.3245 | 1 | 0.5689 |  | 0.0916 | 1 | 0.7622 | 1.4164 | 1 | 0.234 |
|  | 3.7563 | 1 | 0.0526 | 0.0052 | 1 | 0.9424 |  | 0.3611 | 1 | 0.5479 | 2.9683 | 1 | 0.0849 |
|  | 1.8335 | 1 | 0.1757 | 0.102 | 1 | 0.7495 |  | 0.063 | 1 | 0.8018 | 1.3291 | 1 | 0.249 |
|  | 0.2335 | 1 | 0.629 | 0.3603 | 1 | 0.5484 |  | 0.1317 | 1 | 0.7167 | 0.6721 | 1 | 0.4123 |
|  | 0.4699 | 1 | 0.493 | 0.0007 | 1 | 0.9793 |  | 0.6849 | 1 | 0.4079 | 1.3577 | 1 | 0.2439 |
|  | 1.2936 | 1 | 0.2554 | 0.0342 | 1 | 0.8533 |  | 0.0743 | 1 | 0.7852 | 2.4889 | 1 | 0.1146 |
|  | 1.3198 | 1 | 0.2506 | 0.2633 | 1 | 0.6078 |  | 0.1604 | 1 | 0.6888 | 1.1248 | 1 | 0.2889 |
|  | 0.7177 | 1 | 0.3969 | 0.0698 | 1 | 0.7916 |  | 0.7967 | 1 | 0.3721 | 2.7433 | 1 | 0.0977 |
|  | 5.1141 | 1 | 0.0237 | 0.3947 | 1 | 0.5298 |  | 0.4878 | 1 | 0.4849 | 5.068 | 1 | 0.0244 |
|  | 0.8913 | 1 | 0.3451 | 0.8511 | 1 | 0.3563 |  | 1.3776 | 1 | 0.2405 | 1.8582 | 1 | 0.1728 |
|  | 1.2231 | 1 | 0.2688 | 0.0078 | 1 | 0.9296 |  | 1.3237 | 1 | 0.2499 | 1.6533 | 1 | 0.1985 |
|  | 1.9509 | 1 | 0.1625 | 1.8047 | 1 | 0.1791 |  | 1.8645 | 1 | 0.1721 | 5.145 | 1 | 0.0233 |
|  | 5.4563 | 1 | 0.0195 | 0.7342 | 1 | 0.3915 |  | 0.4087 | 1 | 0.5226 | 2.8796 | 1 | 0.0897 |
|  | 3.3209 | 1 | 0.0684 | 1.1907 | 1 | 0.2752 |  | 0.1604 | 1 | 0.6888 | 0.5241 | 1 | 0.4691 |
|  | 7.8296 | 1 | 0.0051 | 0.9094 | 1 | 0.3403 |  | 0.0051 | 1 | 0.9431 | 1.0854 | 1 | 0.2975 |
|  | 5.2184 | 1 | 0.0223 | 0.6052 | 1 | 0.4366 |  | 0.1058 | 1 | 0.7449 | 1.8239 | 1 | 0.1769 |
|  | 3.3209 | 1 | 0.0684 | 2.9089 | 1 | 0.0881 |  | 0.0577 | 1 | 0.8102 | 0.1737 | 1 | 0.6769 |
|  | 2.0075 | 1 | 0.1565 | 1.767 | 1 | 0.1838 |  | 0.3274 | 1 | 0.5672 | 0.6721 | 1 | 0.4123 |
|  | 1.9509 | 1 | 0.1625 | 2.7933 | 1 | 0.0947 |  | 0.027 | 1 | 0.8695 | 1.5087 | 1 | 0.2193 |
|  | 0.1804 | 1 | 0.671 | 0.3822 | 1 | 0.5364 |  | 0.766 | 1 | 0.3815 | 0.6795 | 1 | 0.4098 |
|  | 0.319 | 1 | 0.5722 | 0.0489 | 1 | 0.8251 |  | 0.1897 | 1 | 0.6631 | 0.3766 | 1 | 0.5394 |
|  | 0.1344 | 1 | 0.7139 | 0.1604 | 1 | 0.6888 |  | 0.008 | 1 | 0.9289 | 0.4188 | 1 | 0.5175 |
|  | 0.0099 | 1 | 0.9207 | 0.2446 | 1 | 0.6209 |  | 0.1163 | 1 | 0.7331 | 1.2199 | 1 | 0.2694 |


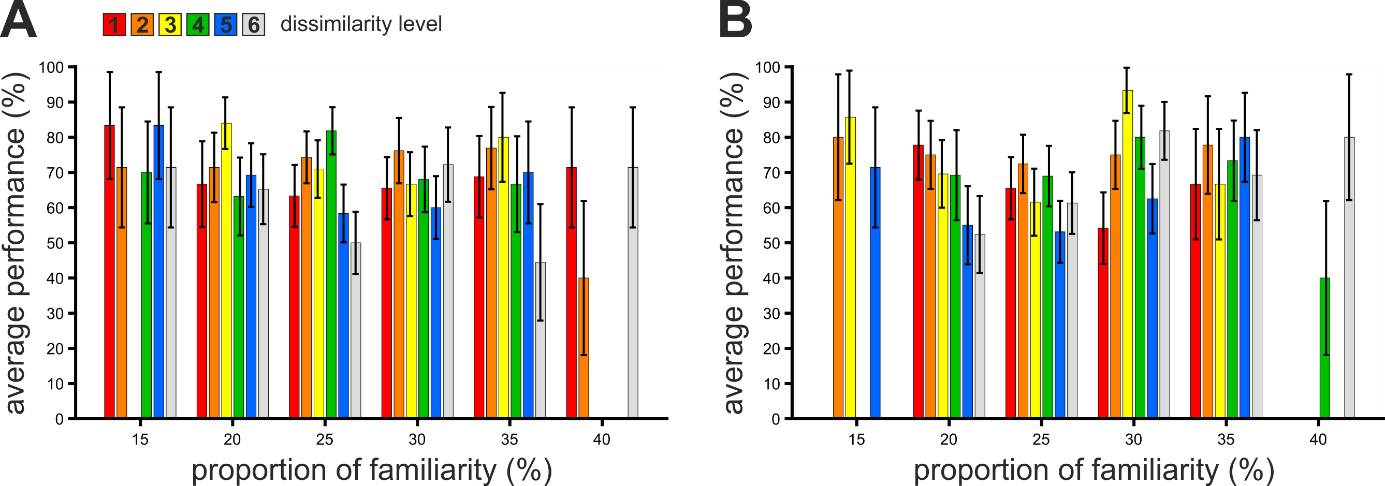


**Figure S3**: Categorization performance in ‘exemplar’ sessions was independent of the overall similarity between sample and all previously encountered stimuli from the same category (i.e., sample and matching choice stimulus). The proportion of familiarity was calculated by counting the number of previously seen stimuli within the same dissimilarity level and dividing it by the total number of stimuli seen until that point (calculation within trials 11 to 30 across all sessions; choice of trials due to: distribution of dissimilarity levels (more trials would result in more uniform distribution and thus less possible differences between familiar and unfamiliar), expectation of largest effect in the early trials, less confound with effects of learning in early trials). The average performance was then calculated across all sessions per dissimilarity level as a function of the proportion of familiarity for the given level. For example, if in trial 11 the sample belonged to L5, we counted all previous occurrences of a L5 stimulus within this category (i.e., as sample and choice) and divided it by the total number of stimuli seen until that point. The average performance was then calculated across all sessions, based on the respective performance with a sample from L5 and the same proportion of familiarity. Bars show average performance ± SEM per dissimilarity level (color-coded). **A** bird 1, n = 60 sessions; **B** bird 2, n = 52 sessions.


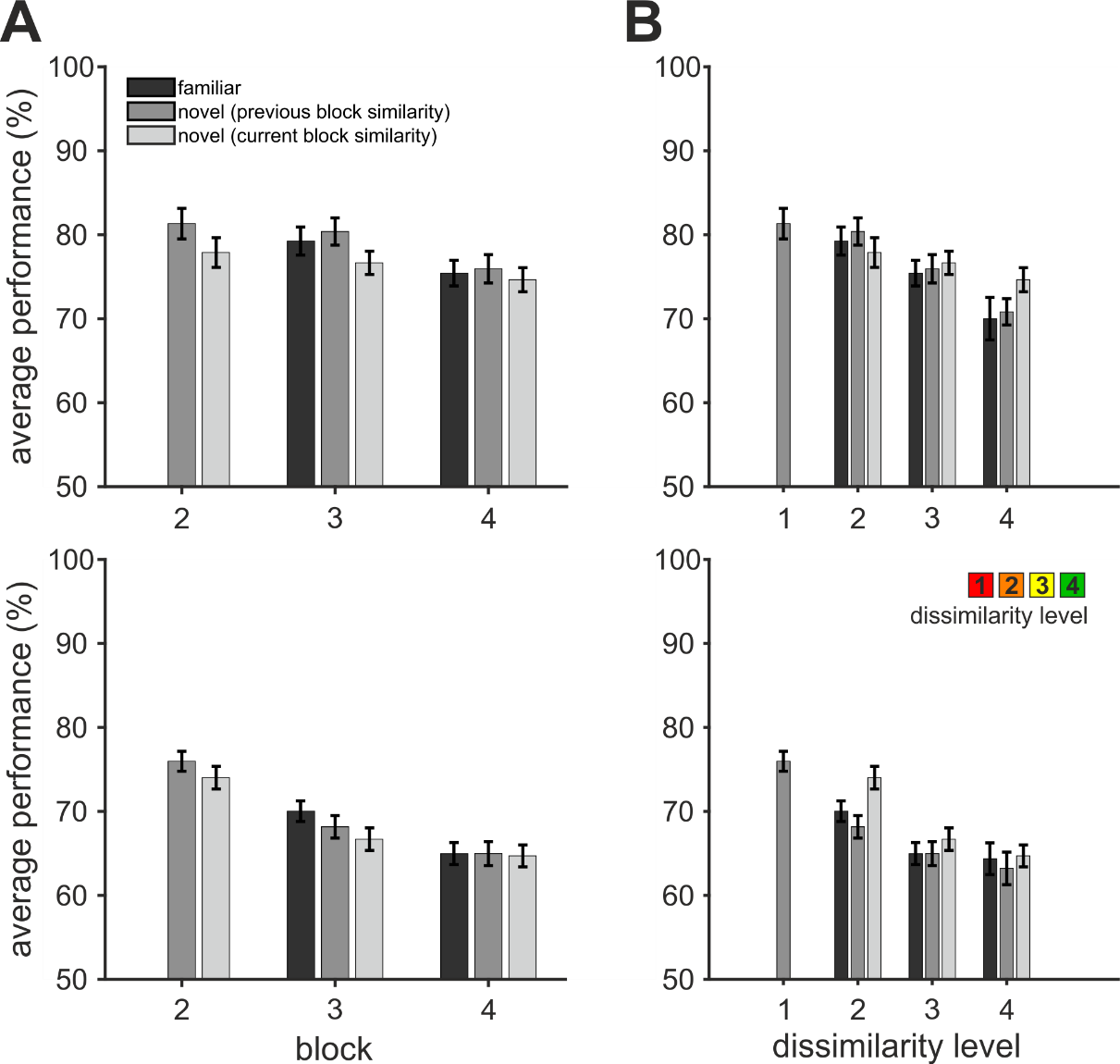


**Figure S4:** Performance per sample familiarity in ‘prototype’ sessions, shown separately per block (**A**, top for bird 1, bottom for bird 2) and sample dissimilarity level (**B**, top for bird 1, bottom for bird 2). Performance was largely similar in trials with familiar and novel sample. Performance decreased strongest due to an increase of sample dissimilarity level. In ‘prototype’ sessions, each full block (starting in block 3) consisted of familiar stimuli from the previous block (familiar, F) and of two types of novel stimuli that the bird had not yet encountered. Half of the novel stimuli had the same dissimilarity level as the familiar stimuli (novel, previous block similarity, NF), the other half had the subsequent dissimilarity level (novel, current block similarity, N). For example, in block 3 the birds encountered F and NF stimuli of dissimilarity level L2, and N stimuli of dissimilarity level L3. We found no significant difference between F and NF stimuli in the ‘prototype’ sessions, for either bird, for any dissimilarity level or any block (Tab. S3). Therefore, as expected, the birds did not memorize individual stimuli in ‘prototype’ sessions but focused on the available prototype instead.

**Table S4:** Overview of statistical results of one-way ANOVAs testing factor familiarity (per block and per dissimilarity level) in ‘prototype’ sessions. Bayesian statistics are given in the last column to report support for the null hypothesis (null) over the alternative hypothesis (fam.). P(M) – prior probability of model; P(M|data) – posterior probability of model, given the observed data; $\mathrm{BF}_{M}$ – posterior model odds; $\mathrm{BF}_{10}$ – Bayes factor for evidence of model to best model. M – tested model, either null, or familiarity (fam).

|  | **group** | **Results** | **Bayesian results** |
| --- | --- | --- | --- |
| Bird 1  block | 2 | F(2,156) = 1.50, p = 0.2271, | P(null) = 0.5, P(null\|data) = 0.787, BF_null_ = 3.684, B_10_ = 1.000 P(fam) = 0.5, P(fam\|data) = 0.213, BF_fam_ = 0.271, B_10_ = 0.271 |
|  | 3 | F(2,156) = 0.18, p = 0.8360 | P(null) = 0.5, P(null\|data) = 0.932, BF_null_ = 13.614, B_10_ = 1.000 P(fam) = 0.5, P(fam\|data) = 0.068, BF_fam_ = 0.073, B_10_ = 0.073 |
|  | 4 | F(2,108) = 0.13, p = 0.8768 | P(null) = 0.5, P(null\|data) = 0.915, BF_null_ = 10.784, B_10_ = 1.000 P(fam) = 0.5, P(fam\|data) = 0.085, BF_fam_ = 0.093, B_10_ = 0.093 |
| Bird 2  block | 2 | F(2,174) = 1.64, p = 0.1968 | P(null) = 0.5, P(null\|data) = 0.791, BF_null_ = 3.794, B_10_ = 1.000 P(fam) = 0.5, P(fam\|data) = 0.209, BF_fam_ = 0.264, B_10_ = 0.264 |
|  | 3 | F(2,174) = 0.01, p = 0.9871 | P(null) = 0.5, P(null\|data) = 0.945, BF_null_ = 17.238, B_10_ = 1.000 P(fam) = 0.5, P(fam\|data) = 0.055, BF_fam_ = 0.058, B_10_ = 0.058 |
|  | 4 | F(2,93) = 2.05, p = 0.1343 | P(null) = 0.5, P(null\|data) = 0.696, BF_null_ = 2.287, B_10_ = 1.000 P(fam) = 0.5, P(fam\|data) = 0.304, BF_fam_ = 0.437, B_10_ = 0.437 |
| Bird 1  dissimilarity level | 2 | F(2,156) = 0.55, p = 0.5778 | P(null) = 0.5, P(null\|data) = 0.919, BF_null_ = 11.342, B_10_ = 1.000 P(fam) = 0.5, P(fam\|data) = 0.081, BF_fam_ = 0.088, B_10_ = 0.088 |
|  | 3 | F(2,156) = 0.16, p = 0.8501 | P(null) = 0.5, P(null\|data) = 0.936, BF_null_ = 14.549, B_10_ = 1.000 P(fam) = 0.5, P(fam\|data) = 0.064, BF_fam_ = 0.069, B_10_ = 0.069 |
|  | 4 | F(2,124) = 1.55, p = 0.2153 | P(null) = 0.5, P(null\|data) = 0.783, BF_null_ = 3.608, B_10_ = 1.000 P(fam) = 0.5, P(fam\|data) = 0.217, BF_fam_ = 0.277, B_10_ = 0.277 |
| Bird 2  dissimilarity level | 2 | F(2,174) = 5.22, p = 0.0063 | P(null) = 0.5, P(null\|data) = 0.168, BF_null_ = 0.202, B_10_ = 1.000 P(fam) = 0.5, P(fam\|data) = 0.832, BF_fam_ = 4.941, B_10_ = 4.941 |
|  | 3 | F(2,156) = 0.53, p = 0.5880 | P(null) = 0.5, P(null\|data) = 0.922, BF_null_ = 11.862, B_10_ = 1.000 P(fam) = 0.5, P(fam\|data) = 0.078, BF_fam_ = 0.084, B_10_ = 0.084 |
|  | 4 | F(2,120) = 0.15, p = 0.8644 | P(null) = 0.5, P(null\|data) = 0.915, BF_null_ = 10.772, B_10_ = 1.000 P(fam) = 0.5, P(fam\|data) = 0.085, BF_fam_ = 0.093, B_10_ = 0.093 |


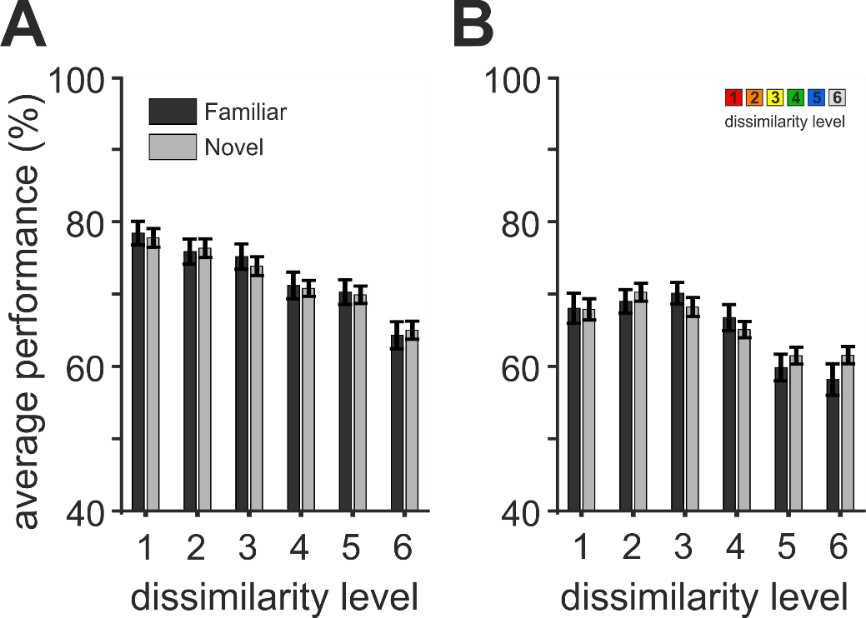


**Figure S5:** Performance per dissimilarity level, split into familiar and novel stimuli in ‘exemplar’ sessions (**A** bird 1, **B** bird 2). Performance across all blocks was separately calculated for trials with familiar (dark grey) and novel (light grey) sample. Performance decreased strongest due to an increase of sample dissimilarity level. In ‘exemplar’ sessions, each full block (starting in block 2) consisted of 1/3 of familiar stimuli and 2/3 of novel stimuli. Results were very similar to ‘prototype’ sessions, revealing a reduced performance due to an increase in dissimilarity level. The birds again showed a substantial effect of dissimilarity level (i.e., similarity relative to the unknown category base stimulus) but no differences between familiar and novel stimuli (Tab. S3). This result emphasizes that the birds did not use the familiarity of stimuli to categorize, even in ‘exemplar’ sessions, but relied on stimulus similarity to the prototype.

**Table S5:** Overview of statistical results of dependent t-test for factor familiarity (per dissimilarity level) in ‘exemplar’ sessions. Bayes factors from a Bayesian paired samples t-test, B_10_ – support for the alternative hypothesis (difference between familiar and novel) B_01_ - support for the null hypothesis (no difference between familiar and novel).

|  | **Dissimilarity level** | **Results** | **Bayes factors** |
| --- | --- | --- | --- |
|  | 1 | t(59) = 0.3637, p = 0.7174 | B_10_ = 0.147, B_01_ = 6.816 |
|  | 2 | t(59) = -0.2582, p = 0.7972 | B_10_ = 0.149, B_01_ = 6.715 |
|  | 3 | t(59) = 0.7767, p = 0.4405 | B_10_ = 0.188, B_01_ = 5.321 |
|  | 4 | t(59) = 0.2049, p = 0.8383 | B_10_ = 0.143, B_01_ = 6.982 |
|  | 5 | t(59) = 0.2091, p = 0.8351 | B_10_ = 0.143, B_01_ = 6.979 |
|  | 6 | t(59) = -0.4001, p = 0.6905 | B_10_ = 0.160, B_01_ = 6.260 |
| Bird 2 | 1 | t(51) = 0.0654, p = 0.9481 | B_10_ = 0.156, B_01_ = 6.410 |
|  | 2 | t(51) = -0.7171, p = 0.4766 | B_10_ = 0.168, B_01_ = 5.952 |
|  | 3 | t(51) = 0.9452, p = 0.3490 | B_10_ = 0.221, B_01_ = 4.532 |
|  | 4 | t(51) = 0.9039, p = 0.3703 | B_10_ = 0.219, B_01_ = 4.558 |
|  | 5 | t(51) = -0.8047, p = 0.4247 | B_10_ = 0.194, B_01_ = 5.167 |
|  | 6 | t(51) = -1.3710, p = 0.1764 | B_10_ = 0.281, B_01_ = 3.556 |


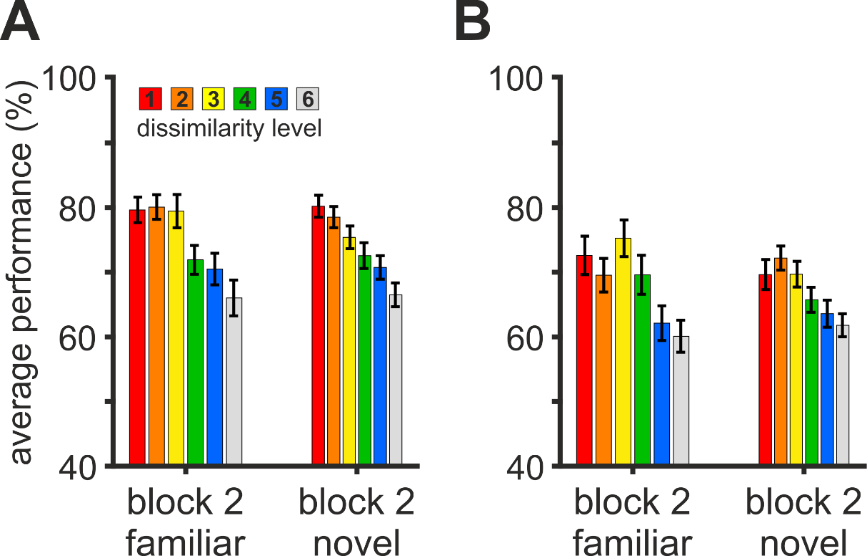


**Figure S6:** Performance in block 2 of ‘exemplar’ sessions, split into familiar and novel sample stimuli. **A** bird 1, **B** bird 2. In ‘exemplar’ sessions, block 2 was the first block in which both familiar and novel stimuli appeared. We found no difference between trials with novel and familiar sample within the same dissimilarity level. None of the direct comparisons within a dissimilarity level (i.e., dissimilarity level L1, familiar vs. novel, etc.) yielded a significant difference between familiar and novel stimuli (Tab. S5).

**Table S6:** Overview of statistical results of dependent t-test for factor familiarity in block 2 of ‘exemplar’ sessions. Bayes factors from a Bayesian paired samples t-test, B_10_ – support for the alternative hypothesis (difference between familiar and novel) B_01_ - support for the null hypothesis (no difference between familiar and novel).

|  | **Dissimilarity level** | **Results** | **Bayes factors** |
| --- | --- | --- | --- |
| Bird 1 | 1 | t(59) = -0.2517, p = 0.8022 | B_10_ = 0.155, B_01_ = 6.464 |
|  | 2 | t(59) = 0.7036, p = 0.4845 | B_10_ = 0.173, B_01_ = 5.771 |
|  | 3 | t(59) = 1.5154, p = 0.1350 | B_10_ = 0.366, B_01_ = 2.732 |
|  | 4 | t(59) = -0.2371, p = 0.8134 | B_10_ = 0.155, B_01_ = 6.442 |
|  | 5 | t(59) = -0.0838, p = 0.9335 | B_10_ = 0.147, B_01_ = 6.824 |
|  | 6 | t(59) = -0.1545, p = 0.8778 | B_10_ = 0.146, B_01_ = 6.871 |
| Bird 2 | 1 | t(51) = 0.9414, p = 0.3509 | B_10_ = 0.237, B_01_ = 4.214 |
|  | 2 | t(51) = -0.9647, p = 0.3392 | B_10_ = 0.256, B_01_ = 3.912 |
|  | 3 | t(51) = 1.6129, p = 0.1129 | B_10_ = 0.460, B_01_ = 2.180 |
|  | 4 | t(51) = 1.0428, p = 0.3020 | B_10_ = 0.237, B_01_ = 4.225 |
|  | 5 | t(51) = -0.4629, p = 0.6454 | B_10_ = 0.165, B_01_ = 6.050 |
|  | 6 | t(51) = -0.5946, p = 0.5547 | B_10_ = 0.174, B_01_ = 5.738 |


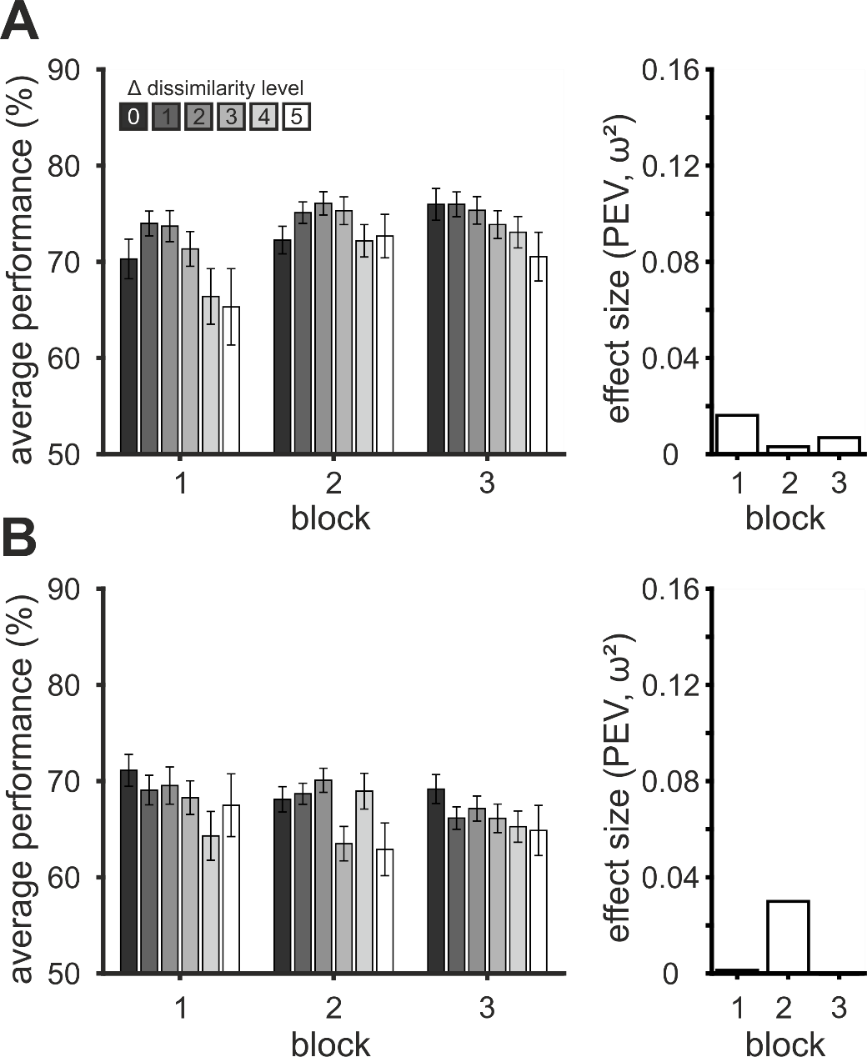


**Figure S7:** Performance per Δ-level per block in ‘exemplar’ sessions. Δ-level was calculated as the absolute difference in dissimilarity level between sample and matching choice stimulus. Performance was significantly affected by Δ-level in bird 1 but not bird 2 (calculated as one-way ANOVA with factor Δ-level per bird). The effect sizes per block revealed no clear pattern. **A** bird 1, n = 60 sessions; **B** bird 2, n = 52 sessions.
